# Circulating eNAMPT in Glaucoma: A Semi-Quantitative Plasma Analysis Before and After Nicotinamide Supplementation

**DOI:** 10.1167/tvst.15.1.37

**Published:** 2026-01-28

**Authors:** Antoni Vallbona-Garcia, Simon T. Gustavsson, Theo G. M. F. Gorgels, James R. Tribble, Carroll A. B. Webers, Hubert J. M. Smeets, Gauti Jóhannesson, Pete A. Williams, Birke J. Benedikter

**Affiliations:** 1 University Eye Clinic Maastricht, Maastricht University Medical Center, Maastricht, Netherlands; 2 Department of Translational Genomics, Maastricht University, Maastricht, Netherlands; 3 Mental Health and Neuroscience Research Institute, Maastricht University, Maastricht, Netherlands; 4 Department of Clinical Sciences, Ophthalmology, Umeå University, Umeå, Sweden; 5 Department of Clinical Neuroscience, Division of Eye and Vision, St. Erik Eye Hospital, Karolinska Institutet, Stockholm, Sweden; 6 Department of Ophthalmology, University of Iceland, Reykjavík, Iceland; 7 Centre for Eye Research Australia, Royal Victorian Eye and Ear Hospital, Melbourne, Australia

**Keywords:** extracellular nicotinamide phosphoribosyltransferase (eNAMPT), nicotinamide, mitochondria, glaucoma, nicotinamide adenine dinucleotide (NAD)

## Abstract

**Purpose:**

Glaucoma is characterized by progressive retinal ganglion cell degeneration. Nicotinamide supplementation has demonstrated neuroprotective potential in glaucoma by raising retinal and optic nerve nicotinamide adenine dinucleotide (NAD) via the salvage pathway, dependent on nicotinamide phosphoribosyltransferase (NAMPT). The NAMPT is essential for retinal function, and its extracellular form (eNAMPT) has been detected in blood. Reduced circulating eNAMPT could indicate impaired NAD biosynthetic capacity and glaucomatous neurodegeneration susceptibility.

**Methods:**

We (i) developed a specific, semiquantitative assay to detect plasma eNAMPT, (ii) explored its potential as biomarker, and (iii) assessed the effect of 2-week nicotinamide supplementation on circulating levels. This was done in samples from a prospective clinical trial at the Eye Clinic, Umeå University Hospital (Sweden), including 30 controls and 90 patients with glaucoma.

**Results:**

A Western blotting assay was designed, detecting eNAMPT (52 kilodalton [kDa]) and transferrin (77 kDa) as housekeeping protein from 0.2 µL EDTA-plasma. Intra- and inter-assay variability were 14.9% and 37.9%, respectively. eNAMPT levels showed no difference between glaucoma and controls, nor changes after supplementation.

**Conclusions:**

eNAMPT is readily and specifically detected by Western blotting in plasma from healthy controls and patients with glaucoma. Given the role of NAD/NAMPT in neurodegeneration, this study provides a platform for specific detection of eNAMPT in liquid biopsies. Further studies specifically designed to study eNAMPT are needed to clarify its role in retinal ganglion cell degeneration and the therapeutic response to nicotinamide.

**Translational Relevance:**

This study established a method to sensitively detect plasma eNAMPT of patients with glaucoma and controls, providing a basis for future biomarker development.

## Introduction

Glaucoma is a group of neurodegenerative diseases hallmarked by the progressive dysfunction and degeneration of retinal ganglion cells (RGCs), the neurons that form the optic nerve. It is the leading cause of irreversible blindness, with more than 80 million people being affected worldwide. Elevated intraocular pressure (IOP) is the most prominent glaucoma risk factor. However, current treatments targeting IOP do not prevent disease progression in all cases, underlining the complex pathology of the disease.^1^^–^^3^ Since RGCs have high cellular energy requirements, it has been proposed that they might be particularly susceptible to bioenergetic impairments, and, therefore, mitochondrial dysfunction.^4^^–^^7^ Novel therapies aiming to boost neuroprotection and survivability through improving the bioenergetic state of the RGCs are being explored, for example, targeting nicotinamide adenine dinucleotide (NAD)-related pathways which have progressed to phase II and phase III clinical trials.^8^^–^^10^

Nicotinamide (NAM), the amide form of vitamin B_3_ and a precursor of NAD, has demonstrated neuroprotective potential for the treatment of glaucoma, and clinical trials with its supplementation are currently ongoing (NCT05275738).^11^^–^^13^ NAM supplementation enhances NAD generation through the NAD salvage pathway.^14^ NAD's primary form, NAD^+^, plays essential roles in maintaining redox balance, supporting biosynthetic pathways, modulating cellular signaling, epigenetic modification, immunity, and DNA repair mechanisms.^14^ Its reduced form, NADH, serves as the primary electron donor for mitochondrial ATP production via the oxidative phosphorylation system (OXPHOS).^9^^,^^13^^–^^17^ Alterations in NAD metabolism have been associated with aging and age-related neurodegenerative diseases.^14^^,^^18^

**Figure 1.**
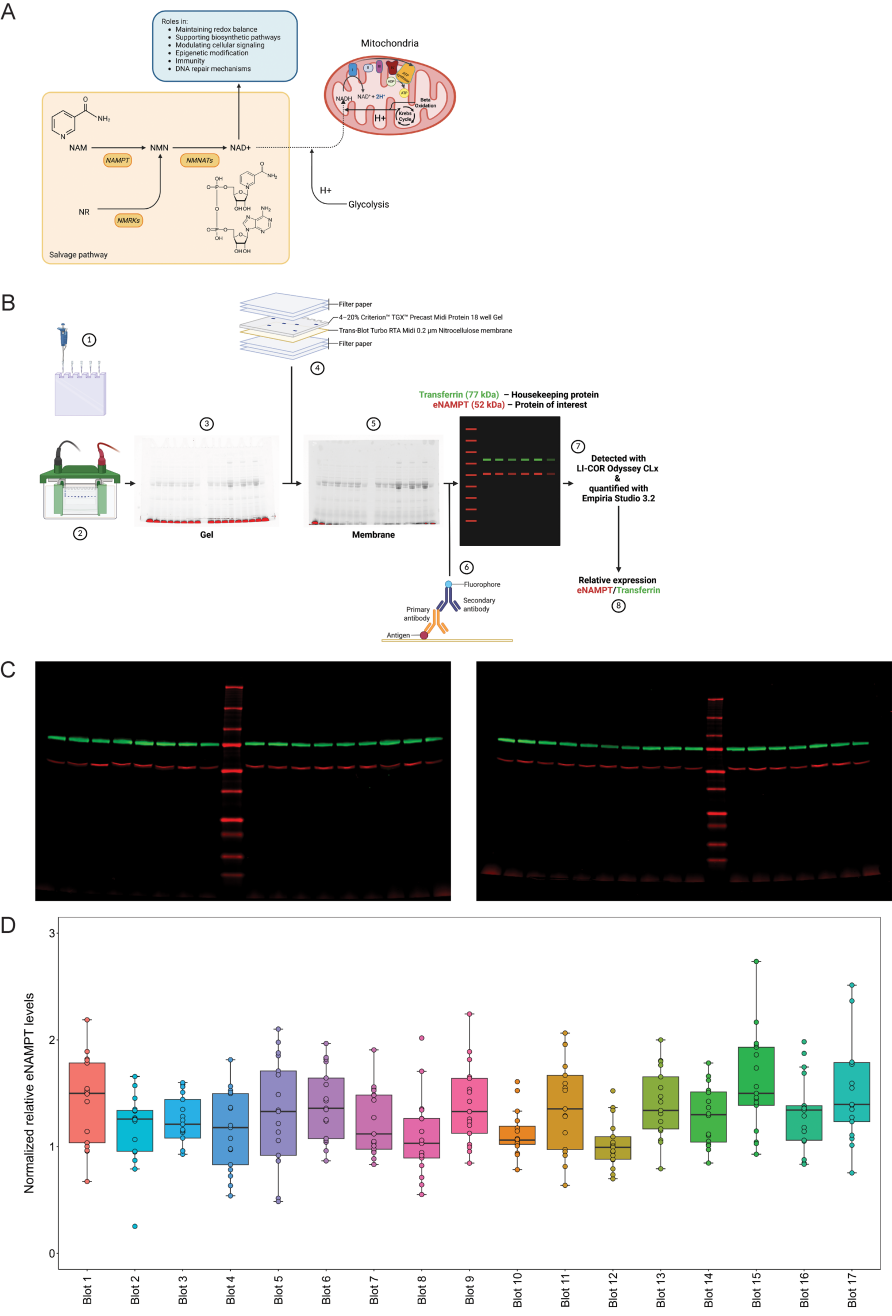
**Summary of the WB assay to measure relative eNAMPT levels in plasma samples.** (**A**) Schematic overview of the NAD salvage pathway, highlighting various cellular roles, including its function as a mitochondrial electron donor in its reduced form (NADH). (**B**) Schematic representation of the final WB workflow. In summary (1) 10 µL of plasma diluted 1:50 in Pierce Lane Marker Reducing Sample Buffer (1×) were loaded into a 4% to 20% Criterion TGX Stain-Free 18 well Protein Midi Gel and (2) run for 45 minutes at 200 V in a Criterion Cell. A reference sample is included in three positions in each gel for further blot corrections. (3) Gel was imaged after electrophoretic separation with Geldoc Go Imaging System for the detection of the Stain-Free technology, allowing the visualization and confirmation of proper protein separation. (4) Gel separated proteins were transferred using the Trans-Blot Turbo Transfer System to a Midi 0.2 µm Nitrocellulose membrane and (5) visualization was again performed to confirm the proper transference and 1 hour blocking was performed at room temperature with slow agitation (approximately 40–45 rpm). (6) Primary antibody (eNAMPT protein of interest; Transferrin Housekeeping protein) overnight incubation following secondary antibody (IRDye 680RD and IRDye 800CW) 1 hour room temperature incubations were performed in black boxes with slow agitation (approximately 40–45 rpm). (7) Dynamic range detection of protein fluorescent bands was performed with the Li-Cor CLx imager and quantified with Empiria Studio 3.2 in order to (8) assess the relative eNAMPT expression to transferrin expression. (**C**) Fluorescence images of two representative Western Blots. (**D**) Distribution of the normalized relative eNAMPT plasma levels measured in all study cohort samples in each of the 17 performed WBs. Normalized relative eNAMPT levels are calculated from the ratio of signals of eNAMPT by transferrin as housekeeping protein and corrected inter-blot wise by the reference sample values. Box and whisker plots indicate the median (*line in box*), 25th and 75th percentiles (*outer lines of box*), and the minimal and maximal values (whiskers). eNAMPT, extracellular nicotinamide phosphoribosyltransferase; NAD, nicotinamide adenine dinucleotide; NAM, nicotinamide; NAMPT, nicotinamide phosphoribosyltransferase; NMN, nicotinamide mononucleotide; NMNATs, nicotinamide-nucleotide adenylyltransferases; NMRKs, nicotinamide riboside kinases; NR, nicotinamide riboside; WB, Western blot.

The synthesis of NAD from NAM in the NAD salvage pathway depends on two enzymatic steps (Fig. 1A). The first step depends on the rate-limiting enzyme nicotinamide phosphoribosyl transferase (NAMPT) to transform NAM into nicotinamide mononucleotide (NMN).^14^^,^^17^ The second step from NMN to NAD depends on the NMNAT1-2 enzymes. NAMPT-mediated NAD biosynthesis has been proposed to be essential for retinal function and vision in mice.^19^ In addition, inhibition of NAMPT enzymatic activity was shown to induce retinal toxicity.^20^ NAMPT is expressed in RGCs, albeit at lower levels than the second enzyme, of which the most predominant isoform in RGCs is NMNAT2.^21^ The expression of NAMPT has been shown to be reduced both in the RGCs of a glaucoma mouse model, as well as part of the optic nerve head (ONH) and the RGC layer of patients with glaucoma, suggesting a decrease in the NAD salvage pathway capacity. Since RGCs highly favor NAD generation via the salvage pathway, a decrease in salvage enzymes might be critical in diseases.^11^^,^^21^

Systemic biomarkers of energy metabolism are increasingly investigated in neurodegenerative disorders because metabolic vulnerability in central nervous system (CNS) neurons can be mirrored by peripheral metabolic signatures.^22^^–^^24^ Given the high energetic demands of RGCs, systemic markers related to NAD biosynthesis may provide a relevant, noninvasive window into bioenergetic stress and neurodegenerative risk in glaucoma. Previous studies have already reported systemic metabolic defects related to NAD metabolism and mitochondrial function in glaucoma.^25^^–^^27^

NAMPT has also been detected in the bloodstream in an extracellular form; eNAMPT (alternatively named Visfatin or pre-B cell colony enhancing factor [PBEF]).^14^^,^^28^^,^^29^ eNAMPT differs from its intracellular form due to post-translational modifications and can be secreted by different cells, for example, cardiac cells, β-cells, and adipocytes via an unknown unconventional pathway. Whereas soluble eNAMPT has originally been suggested to function as a pro-inflammatory cytokine,^28^^,^^30^^–^^35^ more recent research indicates that eNAMPT may also be secreted as catalytically active enzyme encapsulated in extracellular vesicles (EVs).^29^^,^^36^^,^^37^ EV-associated eNAMPT was shown to be taken up by various types of recipient cell types and tissues, including CNS neurons.^29^^,^^32^^,^^36^^,^^38^ More specifically, adipose tissue-specific NAMPT overexpression in mice resulted in both increased circulating eNAMPT and increased in NAMPT and NAD levels in the retina.^29^ This indicates that circulating eNAMPT levels may reflect a systemic capacity to support NAD generation in high-energy demanding tissues such as the retina. Thus, we hypothesized that circulating eNAMPT serves as a systemic biomarker of metabolic vulnerability relevant to glaucomatous neurodegeneration. With current clinical trials ongoing on NAM supplementation in glaucoma, it is also important to study the systemic effects of this supplementation. NAM supplementation aims to increase cellular NAD levels, which in turn activate sirtuins such as SIRT1, whose deacetylation capacity has been proposed to enhance eNAMPT secretion and enzymatic activity, suggesting relevant mechanisms related to NAM therapy.^28^^,^^32^^,^^35^^,^^39^^–^^41^ Due to this mechanistic chain, we hypothesized that we might detect changes in eNAMPT after NAM supplementation.

Although commercial immunoassays such as ELISAs are available to detect circulating NAMPT, substantial variability in specificity and signal interpretation has been reported. Previous studies have shown inconsistent results across assay types and observed discrepancies in detected molecular forms of eNAMPT, highlighting the need for more specific detection methods.^42^

In the current study, we aimed to:

1. Establish a semiquantitative and specific method to measure eNAMPT levels using Western blot (WB).
2. Investigate whether baseline eNAMPT levels differ between different types of glaucoma and healthy controls.
3. Explore whether 2-week oral NAM supplementation alters circulating eNAMPT levels and whether eNAMPT levels are associated with patient phenotypes.

In this study, we demonstrated that eNAMPT is readily available in the blood and can be detected with high specificity and sensitivity in patients with glaucoma and control subjects.

## Materials and Methods

### Study Cohort, Ethical Approval, and Patient Sampling

The cohort has been described in Gustavsson et al., 2023.^43^ In summary, a prospective clinical trial was conducted at the Eye Clinic at Umeå University Hospital, (Umeå, Sweden) which included 30 cases of high tension glaucoma (HTG), 30 cases of normal tension glaucoma (NTG), 30 cases of pseudoexfoliative glaucoma (PEXG), and 30 healthy age and sex-matched controls. During this prospective clinical trial, patients underwent a 2-week accelerated dosing NAM supplementation (1.5*g*/day during the first week and 3 g/day during the second week). Pre- and post-treatment examinations were performed, together with collection of blood. The human studies adhered to the tenets of the Declaration of Helsinki, and the ethics protocols were approved by the Swedish Ethical Review Authority (2020-01525, 2021-01036, 2021-03745, and 2022-04851).

Venous blood samples were collected from each study participant in EDTA tubes at baseline and after NAM treatment, at least 3 hours after the last meal. The tubes were sent immediately after sampling to a centralized biobank (Biobanken Norr) in the same hospital where the samples were collected (University Hospital of Umea). At the biobank, the tubes underwent centrifugation (1500 G, 15 minutes, at room temperature [RT]), and aliquots (all 250 µL) were collected from the tubes using a Hamilton Microlab Star LET. Aliquots of erythrocytes, plasma, and buffy coat were collected from the EDTA tubes. All the aliquots were placed in SBS format cryotubes and then immediately stored at −80°C until further analysis. The time between sampling and storage was 37 to 180 minutes (mean value = 80 minutes and median value = 77 minutes). The aliquots were kept frozen at −80°C as they were sent for analysis. Out of the 120 participants included in the study, the following plasma samples were available and used in this study: 236 plasma samples (*n* = 30 controls, *n* = 30 HTG, *n* = 30 NTG, and *n* = 29 PEXG; 119 pre-NAM/*n* = 30 controls, *n* = 29 HTG, *n* = 29 NTG, and *n* = 29 PEXG; 117 post-NAM). Some samples were not available due to the participant not being able to join the first or second study visit, or the blood fraction could not be properly collected and stored.

### Immunoblotting Experimental Optimization and Design

Optimization of several immunoblotting aspects was performed, which are summarized in Table 1, such as the use of plasma or serum, the sample boiling time, volume of diluted sample to load, antibody concentrations, gel type, incubation protocol, and normalization method. The procedures used for sample preparation and immunoblot execution are described in detail in the following subsections.

**Table 1.** Summary of the Several Parameters That Were Assessed for the Optimization of the Assay on the Detection of Extracellular Nicotinamide Phosphoribosyltransferase (eNAMPT) Protein Levels in Human Plasma

Variable Tested	Conditions Compared	Replicates	Key Observation/Conclusions	Condition Chosen
Sample material	EDTA plasma vs. serum	3 donors	Similar bands signal and morphology	EDTA plasma because eNAMPT was reported in extracellular vesicles (EVs) and serum may contain >90% platelet-derived EVs
Sample heating to 95°C	5 min vs. 30 min	3 donors	While Yoshida et al.^29^ previously reported heating to 95°C for 30 min as requirement for clear eNAMPT bands, eNAMPT signal and band appearance was similar after 5 and 30 min in our hands. Transferrin shows a clear band after 5 min, but smaller and multiple bands at 30 min, reflecting possible degradation products	5 min at 95°C
Sample buffer	Pierce Reducing Lane Marker vs.	3 donors	Similar signal	Pierce Reducing Lane Marker
	Reducing Laemmli Buffer			
Volume of 1:50 diluted plasma	25, 10, 5, and 2.5 µL	Reference sample	10 µL gives clear bands for both eNAMPT and Transferrin without any evidence for fluorescence self-quenching. Fluorescence self-quenching is seen when loading 25 µL, whereas bands become incomplete for 5 and 2.5 µL. The signal increases with increasing loaded volume	10 µL
Dilution NAMPT antibody	1:2500, 1:1000, and 1:500	Reference sample	1:1000 shows a clear, complete, not self-quenched band. Band at 1:2500 and 1:500 show lower and higher signal respectively than 1:1000	1:1000
Dilution transferrin antibody	1:10000, 1:5000, 1:2500, and 1:1000	Reference sample	1:5000 shows a clear, complete, not self-quenched band	1:5000
			1:10000 shows a lower signal than 1:5000	
			1:2500 and 1:1000 show a higher signal than 1:5000	
Gel type	Tris-Glycine extended (Midi, 4–12%, 18 wells, Bio-Rad)/Bolt Bis-Tris (Mini 4–12% 17 wells, Thermo Fisher Scientific)	Reference sample	TGX Midi gels displayed clearer and flatter bands without smear for both proteins, whereas Bolt Bis-Tris showed a strong smile. Better intra-assay variability was also observed on TGX Midi gels across the whole blot (sides and center)	Tris-Glycine extended (Midi, 4–12%, 18 wells)
Antibody incubation protocols	Type of shaking (rolling or flat) and volume of Ab/blocking (10 mL or 20 mL)	Reference sample	Flat incubation, shaking to all sides at ∼40 rpm, with 20 mL of antibody solution in a closed black box gave the most stable less intra-assay variable results on both bands. Rolling incubation and lower volume were linked with higher intra-blot variability/staining gradients	Flat incubation with 20 mL
Normalization measurement	Total protein vs. transferrin	Reference sample	We explored the total protein staining (Stain-Free technology) on the imaged membranes as an alternative method to transferrin as housekeeping normalization protein (see Methods). Quantified total protein did not correlate to the band signals. Possible explanations are (1) high background on total protein images (membrane wet due to transfer buffer) and (2) imaging on a different device (GelDoc Go, Bio-Rad) than fluorescent antibodies (Odyssey CLx, Li-Cor)	Transferrin as housekeeping protein to normalize eNAMPT

The parameters are listed chronologically in the order of testing, and following the parameters were tested after fixing the parameters listed above.

With the optimized protocol, a total of 17 WBs were performed to analyze all 236 plasma samples of the study cohort. Each blot contains samples from six to seven randomized study participants, with the respective treatment paired sample of the subject in the same blot adjacent to it. In all the blots, the same plasma reference sample was situated in three different positions (both extreme sides and the center of the gel) in order to assess the intra and inter-blot variability, and as a correction/normalization factor allowing inter-blot standardization.

### Plasma Sample Preparation for Immunoblot

Plasma aliquots stored at –80°C were altogether thawed overnight (O/N) on ice and inside a cold room (4°C). Plasma was diluted 1:50 in Pierce Lane Marker Reducing Sample Buffer (Thermofisher, 39000; diluted stock from 5 × to 1 × in Milli-Q water). Samples were warmed at 95°C for 5 minutes, cooled immediately back on ice, and then frozen at –80°C.

### Immunoblotting

Per blot, plasma samples in sample buffer were thawed for 10 minutes at RT. Then, the samples were spun down and vortexed prior to loading 10 uL in 4% to 20% Criterion TGX Stain-Free 18 well Protein Gel (Bio-Rad, #5678094). Then, 2 uL of Odyssey One-Color Protein Molecular Weight Marker (Li-Cor, 928-40000), for visible viewing and 700 nm channel near-infrared detection, was also loaded in each gel. Electrophoretic separations were performed at 200 V for 45 minutes using running buffer containing 25 mM Tris, 192 mM glycine, 0.1% SDS, and pH 8.3, in the Criterion Cell (Bio-Rad, 1656001). After electrophoretic separation, gels were imaged with the Geldoc Go Imaging System (Bio-Rad, #12009077) using the Stain-Free Gel mode (45-second activation and 1–3-second exposure) for activation of the Stain-Free technology, confirming the proper protein separation. Then, the proteins were blotted using the Trans-Blot Turbo RTA Midi 0.2 µm Nitrocellulose Transfer Kit (#1704271), according to the manufacturer’s instructions, and the Trans-Blot Turbo Transfer System (Bio-Rad #1704150) with the following MIDI protocol settings: 2.5 A and 25 V for 7 minutes. The blot after transference was imaged with the Geldoc Go Imaging System (Bio-Rad, #12009077) using the Stain-Free Blot mode (1–3-second exposure) to confirm the proper protein transfer.

The blot was blocked for 1 hour at RT in 20 mL of Intercept PBS Blocking Buffer (Li-Cor, 927-70003) diluted 1:2 in PBS 1 × (w/o Ca and w/o Mg), with slow agitation in a plate shaker (approximately 40–45 revolutions per minute [rpm]). After that, 3 washes of 5 minutes each with freshly prepared PBS 1 × (w/o Ca and w/o Mg) with 0.1% Tween were performed. Then, an overnight incubation at 4°C with slow agitation in a plate shaker (approximately 40–45 rpm) was performed with 20 mL of Intercept PBS Blocking Buffer (Li-Cor, 927-70001) diluted 1:2 in PBS 1 × (w/o Ca and w/o Mg), containing primary antibodies with the following dilutions: 1:1000 NAMPT (Adipogen, AG-20A-0034-C100, mouse monoclonal clone OMNI-379) and 1:5000 Transferrin (Abcam, ab82411, rabbit polyclonal).

After the incubation with the primary antibody, membranes were washed 3 times for 5 minutes each with freshly prepared PBS 1 × (w/o Ca and w/o Mg) with 0.1% Tween. Membranes were then incubated at RT for 1 hour in 20 mL of Intercept PBS Blocking Buffer (Li-Cor, 927-70001) diluted 1:2 in PBS 1 × (w/o Ca and w/o Mg) containing goat anti-mouse IRDye 680RD (1:10000, 926-68070) and goat anti rabbit IRDye 800CW (1:10000, 926-32211). Membranes were washed 3 times for 5 minutes each with freshly prepared PBS 1 × (w/o Ca and w/o Mg) with 0.1% Tween and dried 1 hour in filter paper prior to imaging.

### Blot Imaging

Dried membranes were imaged with the Li-Cor CLx imager with the following settings: offset 0.5 mm, lowest quality, scan resolution 169 µm, and autoexposure for both the 700 and the 800 channels. This allowed the detection of both fluorescent antibodies, and of the Odyssey One-Color Protein Molecular Weight Marker in the 700 nm channel.

### Image Analysis

Image analysis was performed with Empiria Studio 3.2 in order to quantify both eNAMPT (protein of interest) and transferrin (housekeeping protein) bands per plasma sample. This is done using the Housekeeping and Targets analysis mode and automatic band detection of the mentioned software.

Quantification of Stain-Free total protein images (.tiff) obtained with the Geldoc Go Imaging System (Bio-Rad, #12009077) after imaging the transferred membrane was performed using ImageJ software 1.54f with an inhouse macro. The mean intensity of the pixels of the portion of bands between 30 and 100 kilodalton (kDa) per plasma sample was quantified. The macro allowed the adjustment of the TIF Stain-Free image to the right angle for the proper quantification and allowed to use the same size box for the quantification of protein and background levels between blots.

### Analysis and Statistics

The normalized relative eNAMPT levels per subject were analyzed using generalized linear mixed-effects modeling (gamma distribution with a log link and Gaussian random effects), including in the fixed effects the study groups (HTG, NTG, PEXG, or control), NAM treatment effect, and the interaction between the treatment and the groups. The model also contained adjustments for phenotypic covariates (sex and age). In the random effects, the variable “Subject” was included in order to pair subjects before and after treatment.

The inference criterion used for comparing models is their ability to predict the observed data, that is, models are compared directly through their minimized minus log-likelihood. When the numbers of parameters in models differ, they are penalized by adding the number of estimated parameters, a form of Akaike information criterion (AIC).^44^ Using the AIC, we found that a generalized linear mixed-effects model with a gamma distribution with a log link and Gaussian random effects fit the normalized relative eNAMPT levels better than a linear model and generalized linear models with other distributions and links.

Possible relations of normalized relative eNAMPT levels in each study group prior to and post-NAM treatment were assessed through Pearson correlations with the disease severity measured through visual field index (%), and optical coherence tomography angiography (OCTA) values from the ONH and the macula. All statistical analysis presented in regard to the normalized relative eNAMPT levels were performed using R software version 4.3.1, and the “glmer” function of the publicly available library “lme4.”^45^^,^^46^

## Results

### Validation of Semiquantitative eNAMPT Detection and Assay Variability

A semiquantitative fluorescent WB assay was designed to detect and quantify the circulating eNAMPT protein levels. Details on the assay optimization are summarized in Table 1. In the final assay setup, 236 human EDTA plasma samples were analyzed on 17 WBs (workflow illustrated in Fig. 1B). Each WB included the same reference plasma sample in three different positions to monitor intra- and inter-blot variability, as well as matched pre- and post-NAM supplementation samples from six to seven randomized study participants from different glaucoma (HTG, NTG, and PEXG) and control groups (see study cohort in the Results section). A compilation of all raw WB can be found in Supplementary Figure S1.

eNAMPT was detected in all samples with a single specific band at 52 kDa (Fig. 1C, see Supplementary Fig. S1), using only 0.2 µL of plasma (10 µL of 1:50 dilution). The abundant plasma protein transferrin was used as housekeeping protein, as common intracellular housekeeping proteins are not suitable for this cell-free sample. Transferrin was also detected in all plasma samples at the same dilution with a band at 77 kDa (see Fig. 1C, Supplementary Fig. S1).

Using the reference sample, we analyzed the intra- and inter-assay variability for raw eNAMPT and transferrin signals, as well as eNAMPT/transferrin ratio. For eNAMPT/transferrin ratio, the intra-assay coefficient of variation (CV) was of 14.9 ± 8.4% and the inter-assay CV was of 37.9%. Detailed information on variability can be found in Supplementary Table S1 and Supplementary Figure S2.

Due to the high inter-assay CV, normalization was performed by dividing each sample's eNAMPT/transferrin ratio by the average eNAMPT/transferrin ratio of the reference sample on the respective blot. The normalized values are referred to as “normalized relative eNAMPT levels,” and these normalized values were used for the analysis of the study cohort. The normalized values of all samples in the study in each WB assay are shown in Figure 1D.

### Study Cohort

The cohort has been described in Gustavsson et al., 2023.^43^ In summary, a prospective clinical trial was conducted at the Eye Clinic at Umeå University Hospital (Umeå, Sweden), with 30 cases of HTG, 30 cases of NTG, 30 cases of PEXG, and 30 healthy controls with similar age and sex distribution included. In this trial, participants underwent a 2-week NAM supplementation (1.5 g/day for the first week and 3 g/day for the second week). Summary of participants phenotypic characteristics can be found in Table 2 and experimental data from this study can be found in Supplementary Dataset S1.

**Table 2.** Study Participants’ Demographics and Clinical Characteristics

	NTG, *n* = 30	HTG, *n* = 30	PEXG, *n* = 30	Control, *n* = 30	*P* Value	*P* NTG Vs. Control	*P* HTG Vs. Control	*P* PEXG Vs. Control	*P* NTG Vs. HTG	*P* NTG Vs. PEXG	*P* HTG Vs. PEXG
Demographic data											
Age, y, mean ± SD	74.5 ± 8.1	74.0 ± 4.2	74.9 ± 5.8	74.0 ± 5.2	0.760	–	–	–	–	–	–
Female sex, *n* (%)	15 (50)	15 (50)	14 (47)	14 (47)	0.988	–	–	–	–	–	–
Ophthalmologic data											
IOP, mm Hg, mean ± SD	11.3 ± 2.5	13.9 ± 3.3	14.1 ± 3.9	13.6 ± 3.6	**0.004** ^*^	0.056	1	1	**0.016** ^*^	**0.008** ^*^	1
MD, dB, mean ± SD	−8.4 ± 5.2	−6.4 ± 4.1	−12.0 ± 7.7	0.3 ± 1.0	**0.008** ^*^	**–**	**–**	**–**	0.431	0.312	**0.006** ^*^
VFI, %, median, IQR	81, 15	88, 21	69, 38	99, 2	**0.009** ^*^	–	–	–	0.453	0.319	**0.007** ^*^
Glaucoma severity,^a^ *n* (%)											
Mild	12 (40)	16 (53)	8 (27)	–	–	–	–	–	–	–	–
Moderate	13 (43)	11 (37)	9 (30)	–	–	–	–	–	–	–	–
Severe	5 (17)	3 (10	13 (43)	–	–	–	–	–	–	–	–
Mean severity, mean ± SD	1.8 ± 0.7	1.6 ± 0.7	2.2 ± 0.8	–	**0.014** ^*^	–	–	–	0.931	0.184	**0.012** ^*^

NTG, normal-tension glaucoma; HTG, high tension glaucoma; PEXG, pseudoexfoliative glaucoma; IOP, intraocular pressure; IQR, interquartile range; MD, mean deviation; VFI, visual field index.

The *P* values in bold represent statistical significance.

Table adapted from Gustavsson et al., 2023.^43^

* *P* value (*P*) < 0.05. ANOVA or Kruskal-Wallis if variable was not normally distributed were performed. Post hoc *P* values were corrected through Bonferroni.

a = According to the simplified Hoddaps classification. Mild glaucoma is seen where the mean deviation (MD) has a value higher of −6 decibels (dB), moderate glaucoma when the MD is between −6 and −12 dB, and severe when the MD is lower than −12 dB.

### Systemic eNAMPT Protein Levels Pre- and Post-NAM Treatment

After quantifying relative eNAMPT plasma levels according to the optimized workflow described above, we moved on to study baseline differences in eNAMPT between the glaucoma groups (HTG, NTG, and PEXG) and healthy controls. We also analyzed the possible within-group effect of the 2-week oral NAM treatment on eNAMPT. The analysis was performed through a paired generalized linear mixed model with study groups, treatment effect, and their interaction (see Methods). The model also included covariate adjustment for age and sex (Supplementary Table S2).

**Figure 2.**
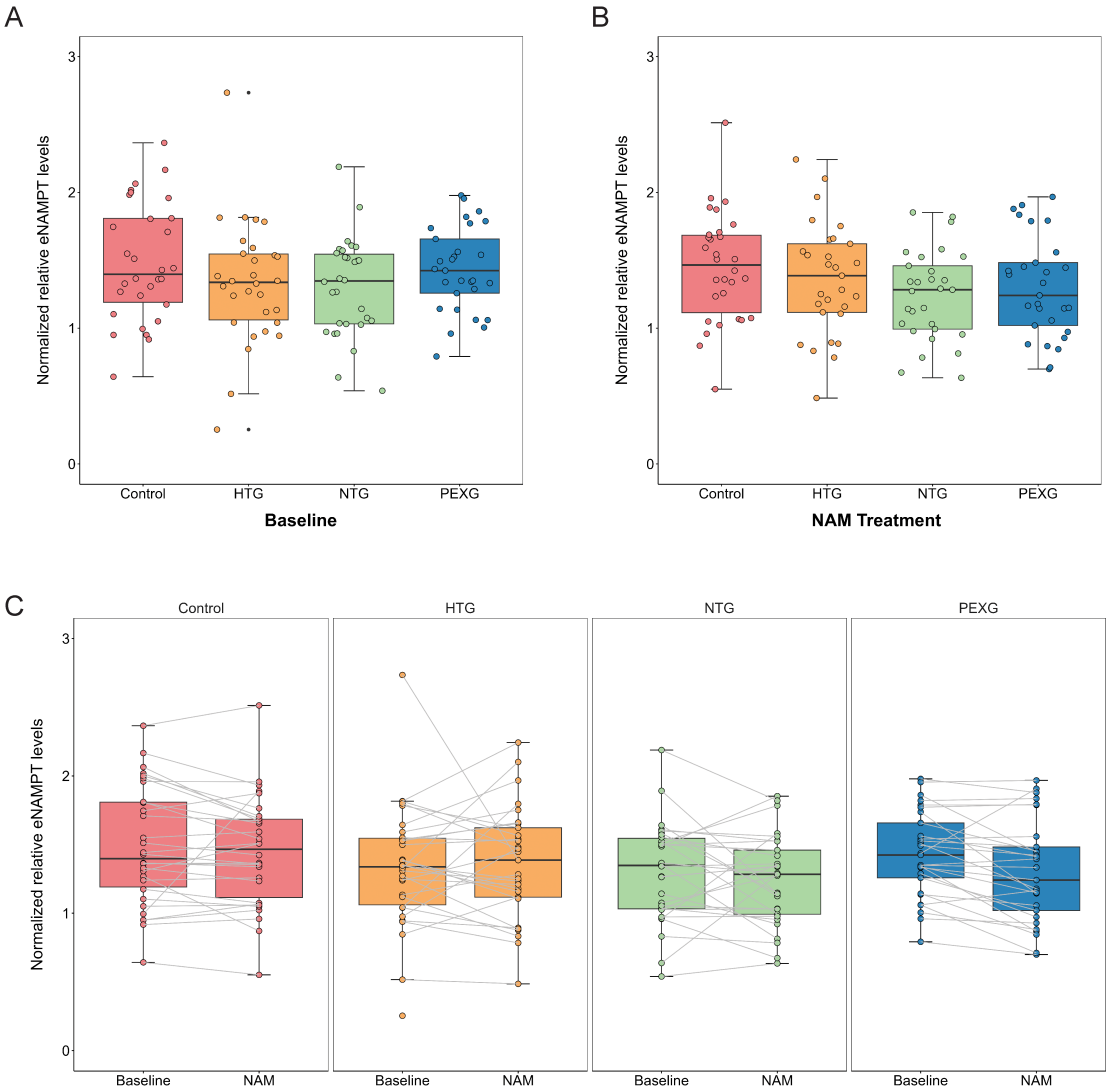
**Normalized relative eNAMPT levels in plasma are comparable between glaucoma subtypes and the control group, and no within-group treatment effect is observed following oral NAM treatment.** (**A, B**) Analysis through a paired generalized linear mixed-model with covariates (age and sex) showed no differences in the normalized relative eNAMPT plasma levels between glaucoma subtypes and controls at baseline (pre-NAM treatment) **A** or after NAM treatment (post-NAM treatment) **B**. (**C**) No significant within-group effect of NAM treatment on normalized relative eNAMPT levels in plasma was observed in the paired generalized linear mixed-model with covariates (age and sex). Normalized relative eNAMPT levels are calculated from the ratio of signals of eNAMPT by transferrin as housekeeping protein and corrected inter-blot wise by the reference sample values. Box and whisker plots indicate the median (*line in box*), 25th and 75th percentiles (*outer lines of box*), and the minimal and maximal values (whiskers). eNAMPT, extracellular nicotinamide phosphoribosyltransferase; HTG, high tension glaucoma; NAM, Nicotinamide; NTG, normal tension glaucoma; PEXG, pseudoexfoliative glaucoma.

**Figure 3.**
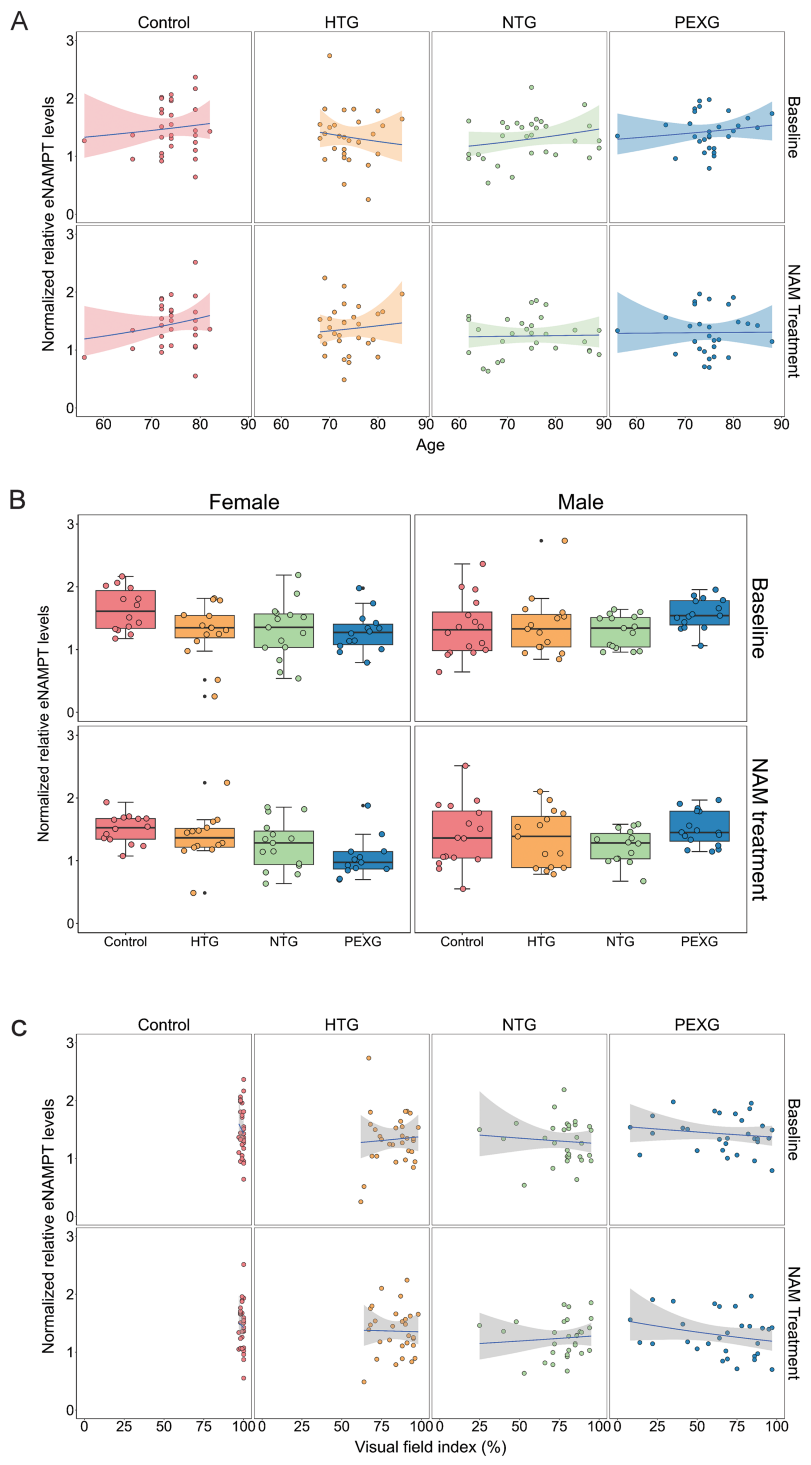
**Normalized relative eNAMPT levels in plasma and its relation with covariates age and sex, and with glaucoma severity measured through visual field index (%).** (**A**) Analysis through a paired generalized linear mixed-model with covariates (age and sex) showed no relation of the normalized relative eNAMPT plasma levels with age in general nor in specific groups, at baseline (pre-NAM treatment) or after NAM treatment (post-NAM treatment). (**B**) No significant relation between normalized relative eNAMPT plasma levels and sex in the mentioned mixed-model was observed, although a tendency for lower levels in glaucoma females than control female subjects was observed. (**C**) No relation between relative eNAMPT levels and glaucoma severity measured by visual field index (%) pre- and post-NAM treatment was observed. Normalized relative eNAMPT levels are calculated from the ratio of signals of eNAMPT by transferrin as housekeeping protein and corrected inter-blot wise by the reference sample values. In **A** and **C** the regression line is plotted together with a shaded area indicating the 95% confidence interval. In **B**, the box and whisker plot indicate the median (*line in box*), 25th and 75th percentiles (*outer lines of box*), and the minimal and maximal values (whiskers). eNAMPT, extracellular nicotinamide phosphoribosyltransferase; HTG, high tension glaucoma; NAM, Nicotinamide; NTG, normal tension glaucoma; PEXG, pseudoexfoliative glaucoma.

We did not observe significant differences in eNAMPT levels between the glaucoma groups and the healthy controls at baseline (Fig. 2A, Supplementary Table S3). After NAM treatment, eNAMPT levels also remained similar between the groups (Fig. 2B, see Supplementary Table S3). Regarding a possible effect of oral NAM treatment on eNAMPT levels, we did not observe a significant change within the study groups (Fig. 2C, see Supplementary Table S3). In relation to covariates, no significant relationship was observed between eNAMPT levels and age or sex, either generally or within specific study groups (Figs. 3A, 3B). However, within sexes, we observed that women from all three glaucoma groups presented a tendency for lower eNAMPT levels in comparison to female controls (see Fig. 3B, Supplementary Table S3). This is similarly observed at baseline and after NAM treatment, and it is not seen in male patients. Finally, we did not observe a relation of eNAMPT levels with glaucoma severity measured through visual field index (%; Fig. 3C).

### eNAMPT Levels do not Correlate With OCTA Parameters

We also investigated the potential relationship between eNAMPT levels and retinal vascular parameters measured by OCTA in either the ONH or the macula. However, no association was observed between eNAMPT levels and OCTA values in either patients with glaucoma or controls, at baseline or following NAM treatment (Supplementary Figs. S3, S4).

## Discussion

In the current study, we established a semiquantitative, specific and sensitive method to detect eNAMPT in plasma and investigate its levels pre- and post-NAM supplementations in glaucoma and corresponding controls. NAMPT is the rate-limiting enzyme of the NAD salvage biosynthetic pathway, which has been proposed to be crucial for retinal function. Circulating blood eNAMPT levels have been proposed to be able to modulate NAD biosynthesis throughout the body, including the retina. Therefore, systemic eNAMPT levels might reflect the bioenergetic capacity of high-energy-demanding cells, such as RGCs, with impaired levels indicating defects in the NAD biosynthetic salvage pathway and thus serving as a marker of metabolic vulnerability. Treatments, such as NAM supplementation, that boost NAD biosynthesis through the NAD salvage pathway are arising as complementary therapies in glaucoma, aiming to improve RGC bioenergetics. Thus, it is also important to study the effects of NAM supplementation on NAD biosynthetic pathways.

### Technical Aspects of eNAMPT Detection by WB

We have used WB to analyze eNAMPT in EDTA plasma, whereas earlier eNAMPT studies have generally used ELISA either performed in serum or plasma.^47^^–^^52^ The reason for choosing WB is that inconsistent results have been reported in a direct comparison of different immunoassay types, probably due to discrepancies in the detection of molecular forms of eNAMPT.^42^ WB offers a higher specificity for eNAMPT detection by enabling the detection of the protein of interest at the correct molecular weight. However, WB technical aspects, for example, protein electrophoresis, protein transfer to a membrane, blocking, antibody binding, and quantification of fluorescence signals from protein bands, lead to a rather high technical variability. This variability was reduced during protocol optimization, and a threshold of 15% intra-blot CV was reached across several WB performed. Although WB allows specific protein detection by size, the throughput is limited. This led to the use of single technical replicates in order to assess the 236 plasma samples in a total of 17 WB assays.

### Association Between eNAMPT and Glaucoma

The amount of eNAMPT in plasma was similar between controls and the three glaucoma subtypes, HTG, NTG, and PEXG, at baseline. The absence of differences in circulating eNAMPT challenges our initial hypothesis that individuals with glaucoma might have lower systemic eNAMPT levels, resulting in reduced delivery of enzymatically active eNAMPT to the retina. However, different forms of eNAMPT (monomeric and dimeric, EV encapsulated and soluble) exist that are not distinguished by our detection method.^29^^,^^53^ If the biologically relevant fraction represents only a small subset of total circulating NAMPT, group differences in active eNAMPT could remain undetected. Another explanation could be that our assay might lack the resolution to detect subtle quantitative differences with the current sample size, as the study was not powered for the assay's variability.

Although eNAMPT may not serve as a standalone biomarker, it could be integrated into multimodal biomarker pipelines that combine, for example, systemic metabolic markers, NAD-related metabolites, and functional mitochondrial measures, such as, in peripheral blood cells. Such composite profiles may better capture bioenergetic vulnerability relevant to RGC survival and may be particularly informative in clinical trials of NAD-enhancing therapies.

With regard to phenotypic traits, no relation of eNAMPT with age was observed. Previous studies showed a decline with age in humans, which might be explained by the age range including younger donors (approximately ∼40 to 80 years old) in comparison to those in this study (56 to 89 years old).^29^ With regard to sex, no relation was observed with eNAMPT levels, whereas sample size limited meaningful statistical subgroup analysis, visual inspection of data showed that female controls had higher eNAMPT levels than male controls. In addition, eNAMPT levels in female patients of all 3 glaucoma groups were at least 16% lower than in female controls. Previous studies that measured eNAMPT levels in serum through ELISA have reported sex differences. In these studies, diurnal variation of eNAMPT levels and a correlation with body weight were also observed.^54^^,^^55^ Taken together, these observations raise the possibility that eNAMPT may have sex-specific effects in glaucoma, as well as sex-specific biomarker potential, perhaps being informative primarily in female patients. Future studies should therefore be designed and powered to evaluate sex-related differences and should control for confounders such as body mass index (BMI) and the timing of blood collection.

Whereas this was the first study to investigate eNAMPT in the context of glaucoma, Kaja et al.^47^ previously assessed eNAMPT as a biomarker in subjects with a history of retinal vascular occlusion, although with a different sample type and method. In these subjects, reduced serum eNAMPT levels, measured through ELISA, were observed. The authors suggested that low bloodstream levels of eNAMPT might indicate low intracellular levels and activity, leading to a reduced endogenous protection against ischemic events.^47^ Vascular pathological situations are also suggested to happen in glaucoma. The vascular theory in glaucoma proposes that diminished perfusion pressure, impaired vascular autoregulation, and disrupted neurovascular coupling contribute to the RGC, and, therefore, optic nerve degeneration.^56^^,^^57^ In the current glaucoma cohort, Gustavsson et al.^43^ previously observed impaired retinal vasculature parameters in the ONH and macula in patients with glaucoma in comparison to controls. However, we found no correlation between eNAMPT and these retinal vascular outcomes. As mentioned, our results suggest that if eNAMPT levels in blood can influence retinal capacity to synthesize NAD, this would not be affected in glaucoma. The NAD salvage pathway enzymatic machinery has been observed to be completely expressed in single-cell and single-nucleus RNA-sequencing (RNA-seq) datasets from postmortem retinas, specifically in RGCs.^21^ This shows the capacity of the cells to independently execute this pathway, in line with a potentially limited importance of eNAMPT for the bioenergetic status of RGCs. Although NAMPT has been shown to be reduced in glaucoma retinal tissue from late-stage patients in the same study, this might only suggest the loss of neuronal tissue in the retina and ONH due to severe disease.

### Forms of eNAMPT and Their Function

In animal studies, increased eNAMPT in the bloodstream has been observed to correlate with an increase in NAD levels in organs such as the hypothalamus or the retina.^29^^,^^54^ The mechanism of how eNAMPT is extracellularly secreted, is internalized by cells, and is able to influence their salvage pathway capacity is not fully known. However, it is suggested to occur only with NAMPT that is encapsulated and delivered in EVs.^29^^,^^36^ eNAMPT was first described as adipokine named Visfatin/ PBEF, having diverse functions in tissues and disease, and being secreted by different cells such as adipocytes or cardiac cells.^30^^–^^34^ For instance, its interaction with certain receptors, such as Toll-like receptor 4 (TLR4), triggering pro-inflammatory cascades, and its relation to vascular inflammation through ROS-dependent NF-κB activation have been studied.^58^^–^^63^ It might be possible that only EV-encapsulated dimeric eNAMPT has catalytic activity and can be functionally delivered to recipient tissues, including the retina, contributing to inter-tissue NAD support.^29^^,^^53^ Our assay quantified total plasma eNAMPT, and distinguished neither EV-encapsulated and soluble, nor different possible forms (dimeric and monomeric). As our WB sample buffer contained 5% SDS, breaking EV membranes and thereby assessing proteins outside and within EVs.^64^ In addition, the WB sample buffer contained reducing agent, breaking up disulfide bonds and thereby separating potential 100 kDa eNAMPT dimers into 52 kDa monomers. In the future, it could be further investigated whether different forms of eNAMPT, or their ratios, have distinct biomarker potential and functional activity in the context of glaucoma, as compared to unencapsulated eNAMPT.

### Effect of NAM Supplementation on eNAMPT

NAM supplementation aims to increase NAD production. The NAD formed performs critical functions at different levels in the cell.^14^ Increased NAD^+^ levels have been shown to activate sirtuins, NAD^+^-dependent histone deacetylases (HDACs), and specifically SIRT1. The SIRT1 can directly interact and influence the activation of PGC1-α, a master regulator of mitochondrial biogenesis.^39^^–^^41^^,^^65^^,^^66^ Interestingly, SIRT1 has been observed to deacetylate NAMPT in its lysine 53, which is suggested to facilitate its release as well as increase the activity of the enzyme.^28^^,^^32^^,^^35^ Therefore, NAM supplementation could lead to an increase in NAD pools, activating SIRT1, and, therefore, promoting the secretion of NAMPT. However, administering NAM supplements orally for 2 weeks did not lead to a change in circulating eNAMPT. Main sources of eNAMPT in the bloodstream are suggested to be cardiac cells, β-cells, and adipocytes.^30^^–^^34^ The mentioned cells are not disease-affected in glaucoma, and their status might be optimal with eNAMPT secretion not being further enhanced by an increase in NAD. Nevertheless, as mentioned in the previous paragraphs, our assay cannot distinguish the possibility of eNAMPT being soluble or EV-associated. Thus, it might be possible that NAM differentially influences eNAMPT secretion depending on its possible form, which is, in our case, masked by the measurement in bulk in plasma.

### Limitations

The relatively high variability of the WB technique may interfere with detection of mild inter-group differences. Second, the technique does not distinguish two (functionally) distinct forms of eNAMPT, within and outside of EVs. Third, the clinical study was not initially designed for our research aims. The duration of NAM supplementation was short and the limited sample size per subgroup may have prevented the detection of differences related to phenotypic traits, for example, sex-specific differences in eNAMPT between patients with glaucoma and controls.

## Conclusions

In conclusion, eNAMPT is readily and specifically detected by WB in EDTA plasma from controls and patients with glaucoma. Given the role of NAD/NAMPT in neurodegenerative diseases, this study provides a platform for the specific detection of eNAMPT in liquid biopsies. Further studies specifically designed to study eNAMPT are needed to clarify its role in RGC degeneration and the therapeutic response to NAM.

## Supplementary Material

Supplement 1

Supplement 2

Supplement 3

Supplement 4
