# Supplementary material for: Circulating eNAMPT in Glaucoma: A Semi-Quantitative Plasma Analysis Before and After Nicotinamide Supplementation
Source: Transl Vis Sci Technol. 2026 Jan 28;15(1):37. doi: 10.1167/tvst.15.1.37 (PMC12859715; doi:10.1167/tvst.15.1.37)

Supplementary figure 1. Compilation of all Western Blots. Includes total protein signal on each of the 17 gels after electrophoresis or membranes after protein transfer, as well as fluorescent antibody staining for transferrin (77 kDa, green) and NAMPT (52 kDa, red). The reference sample positions are marked with a red *. Ab: Antibody; eNAMPT: extracellular nicotinamide phosphoribosyltransferase.

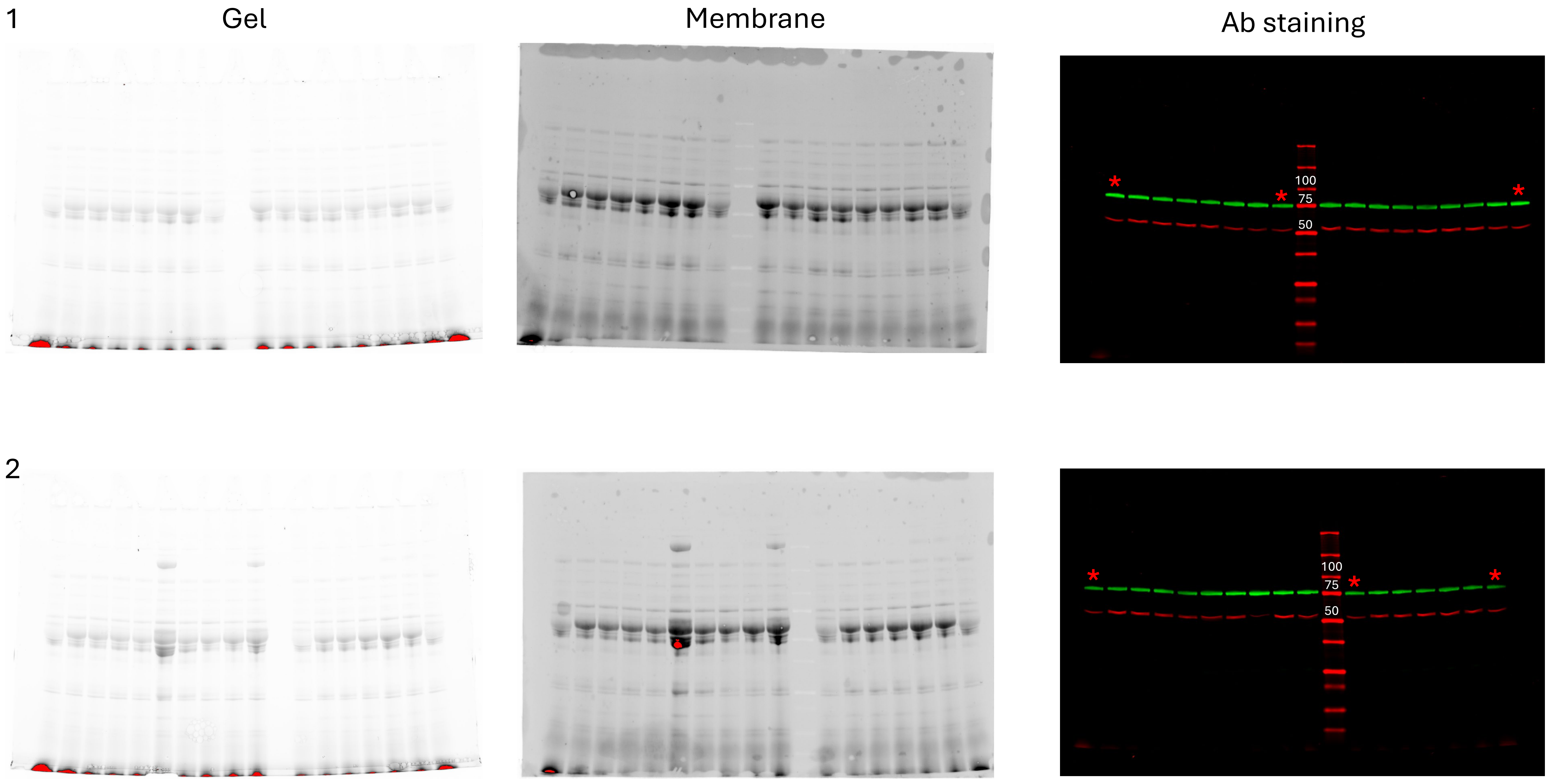
1
Gel
Membrane
Ab staining
100
75
50
2
100
75
50

3 Gel Membrane Ab staining

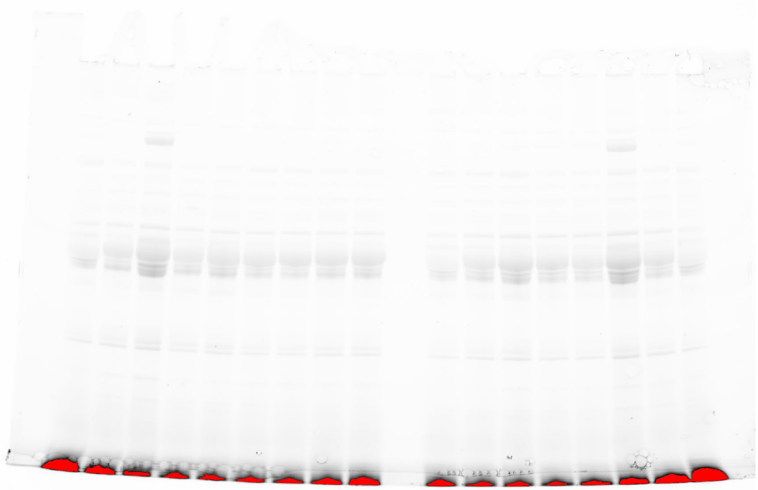

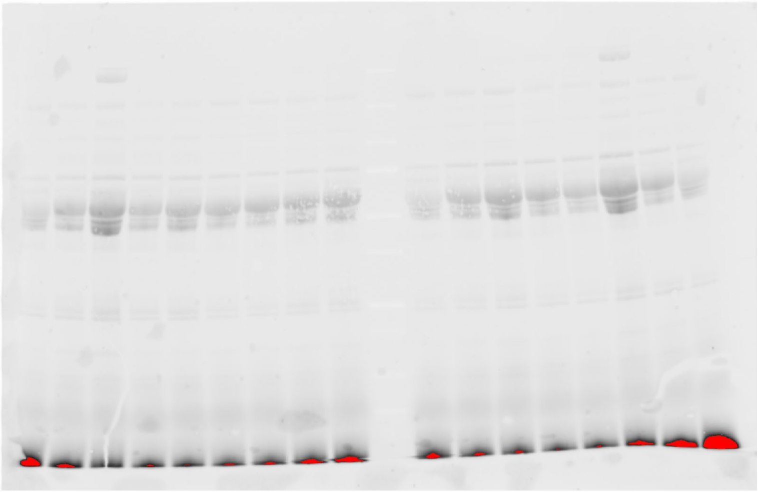

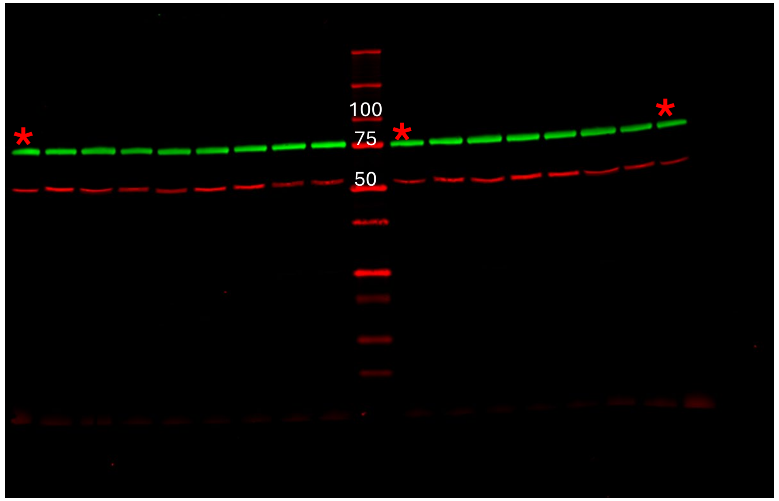

4

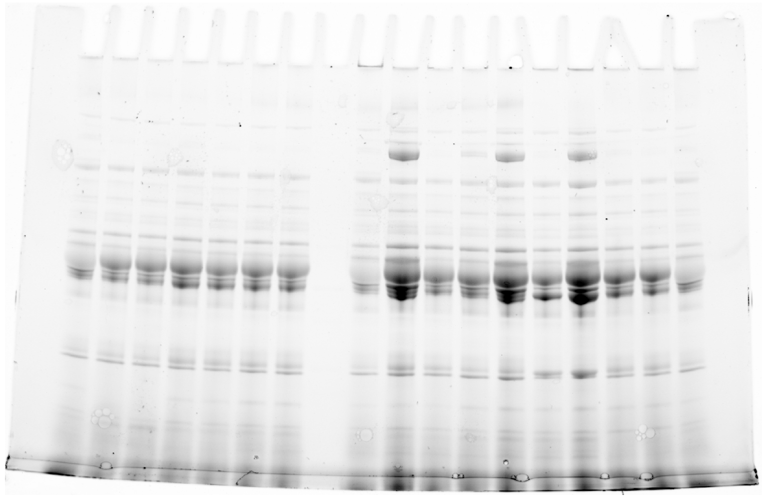

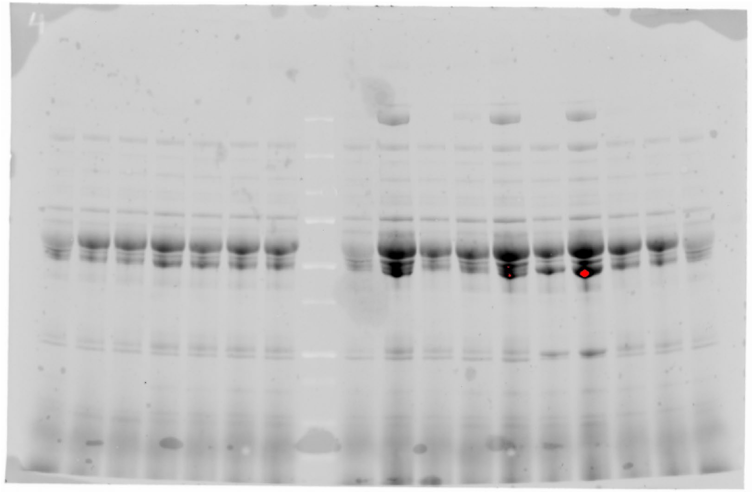

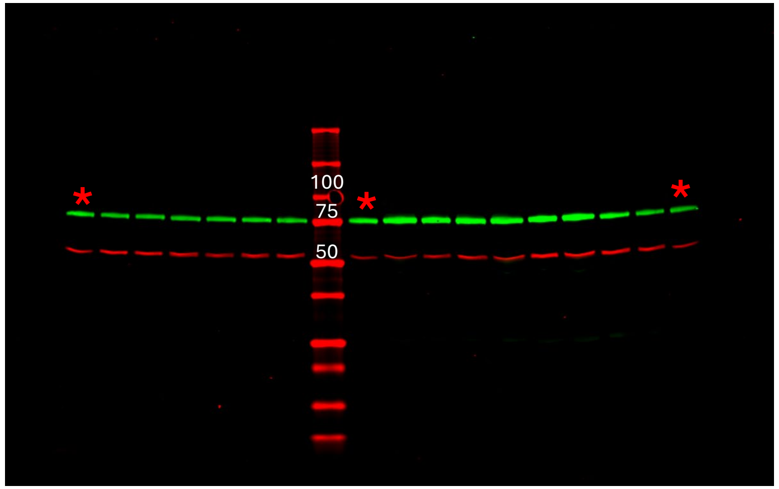

5

Gel

Membrane

Ab staining

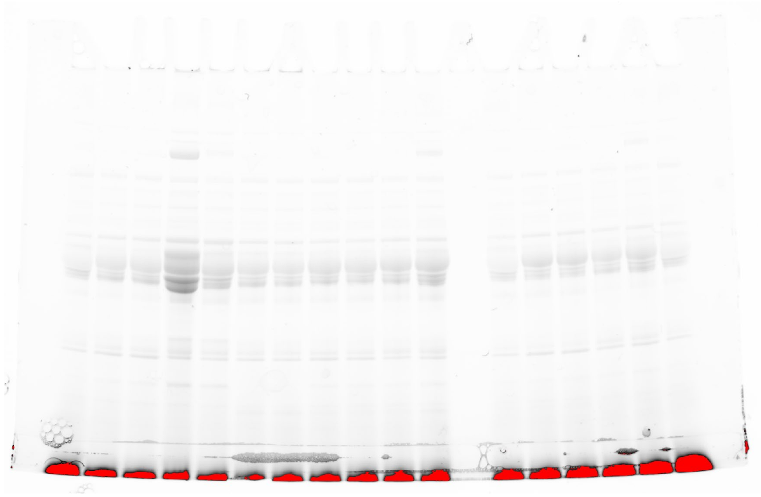

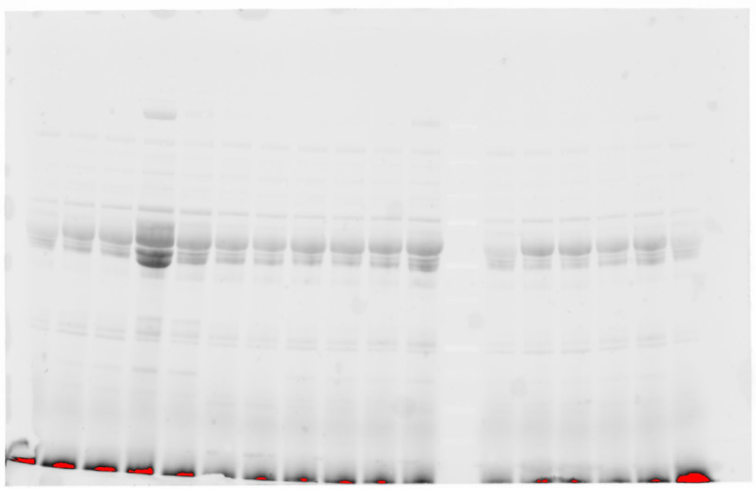

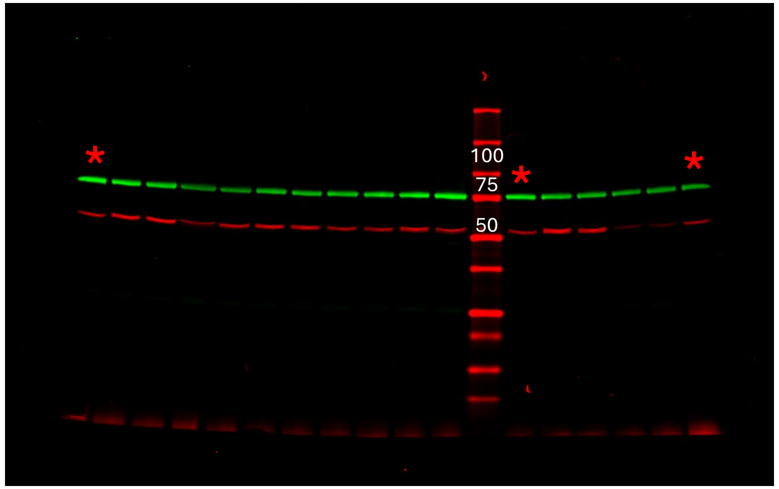

6

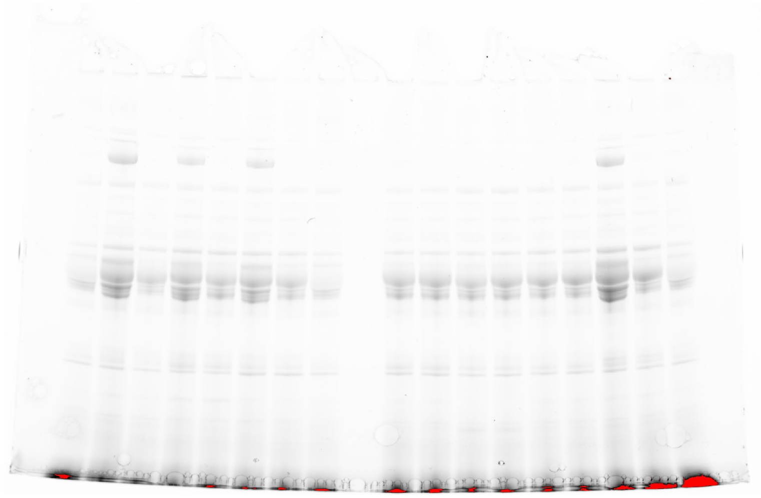

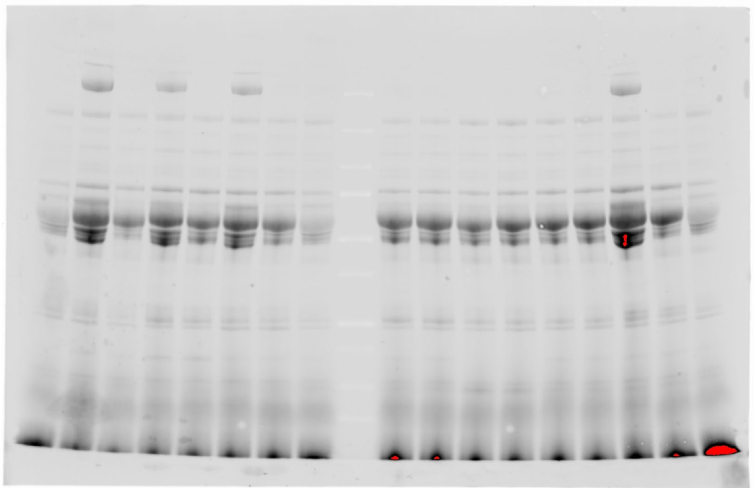

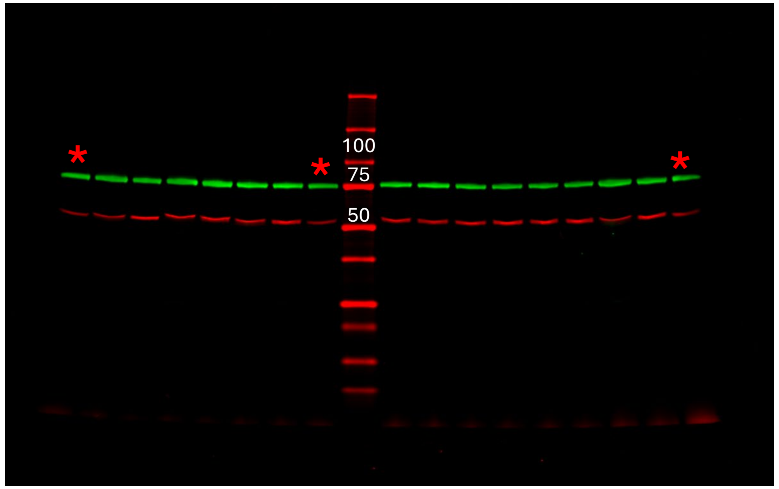

7 Gel Membrane Ab staining

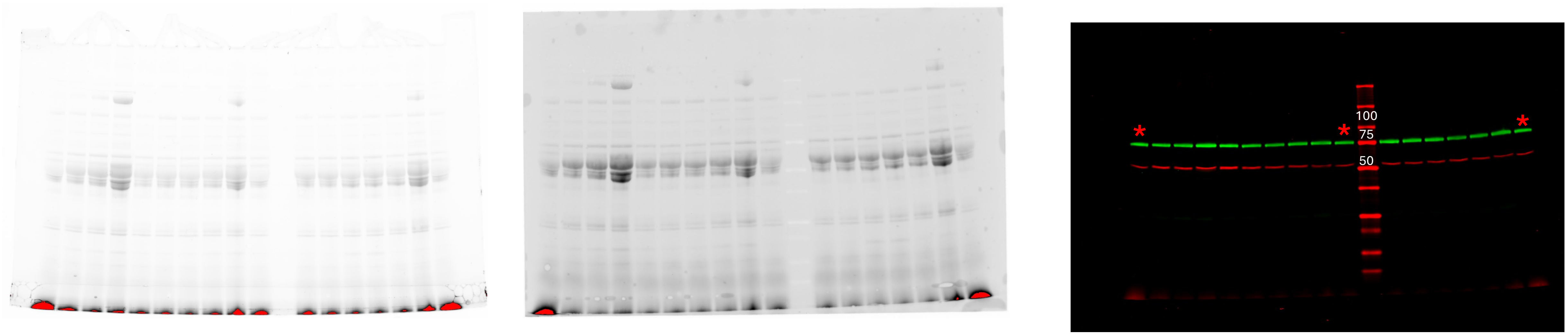

8

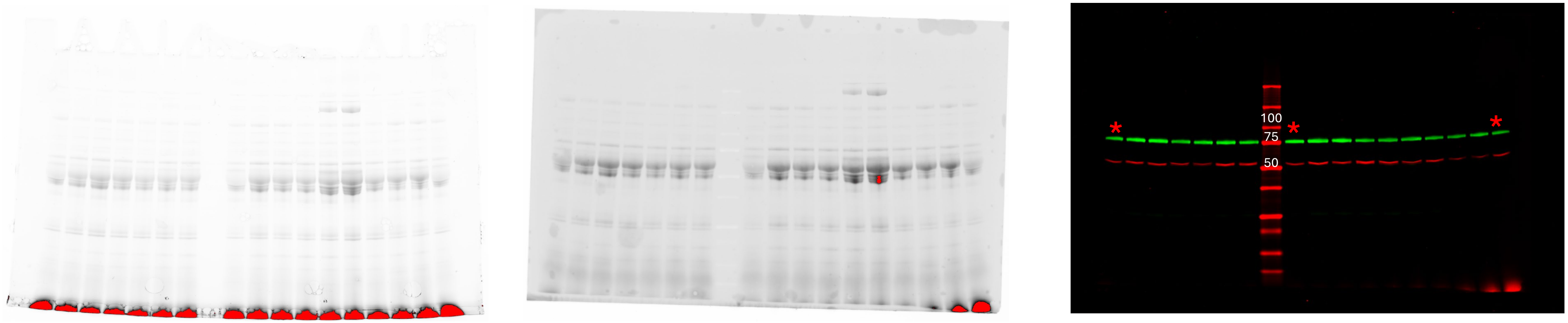

9 Gel Membrane Ab staining

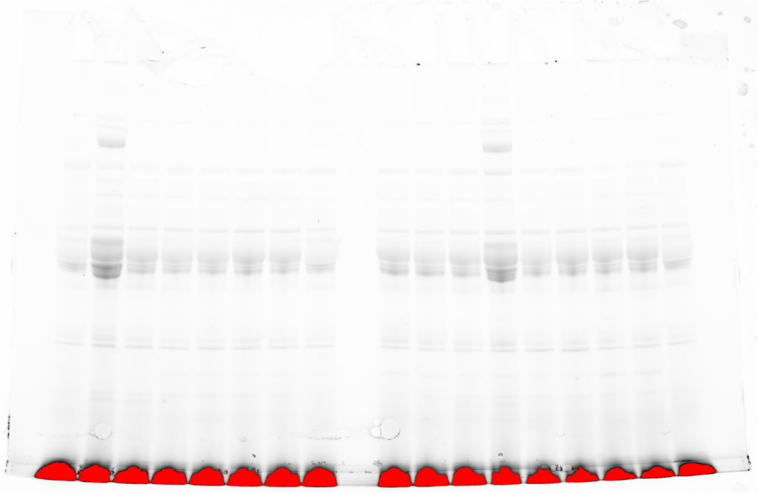

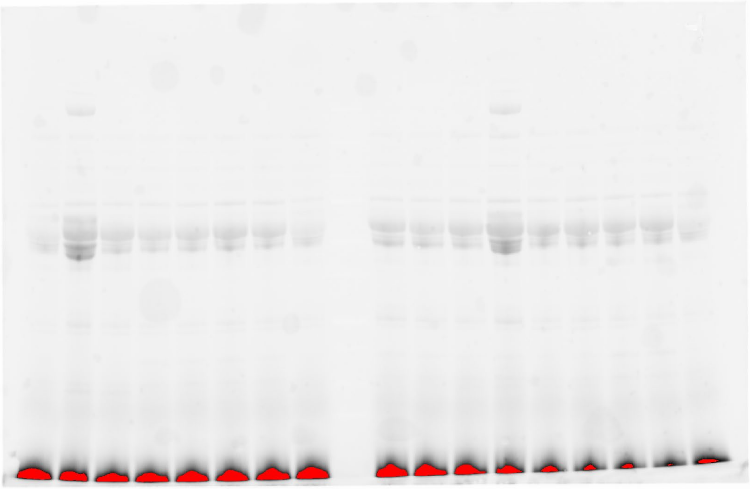

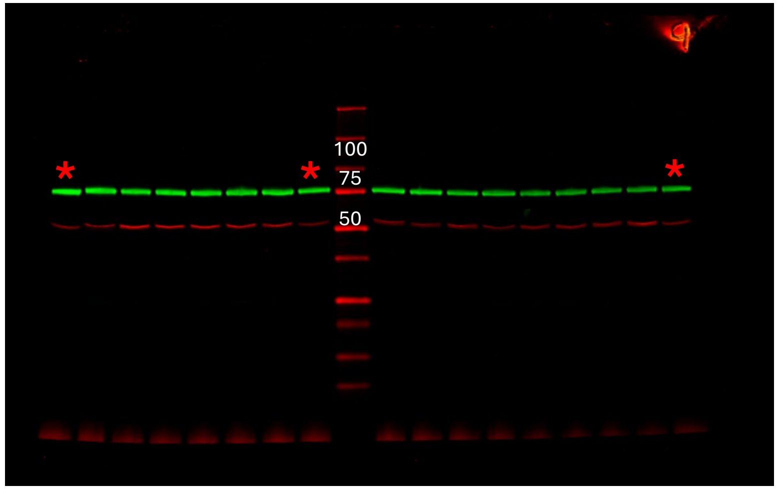

10

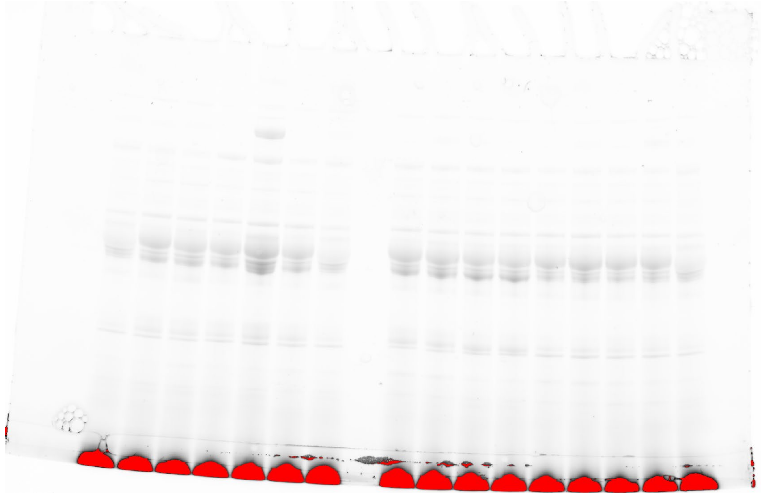

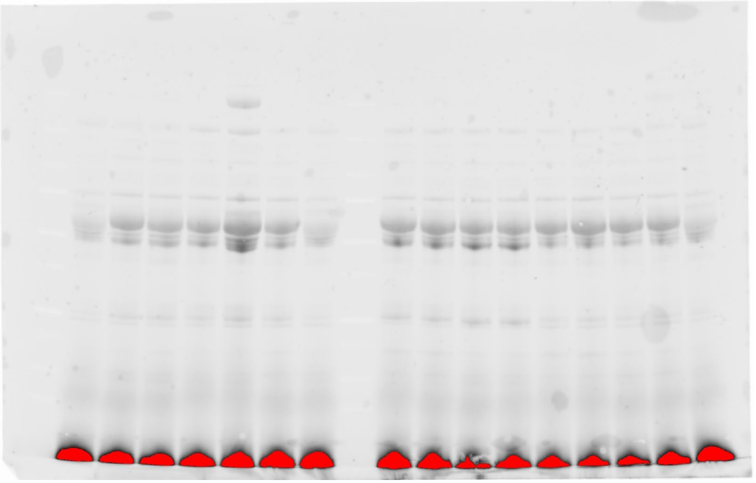

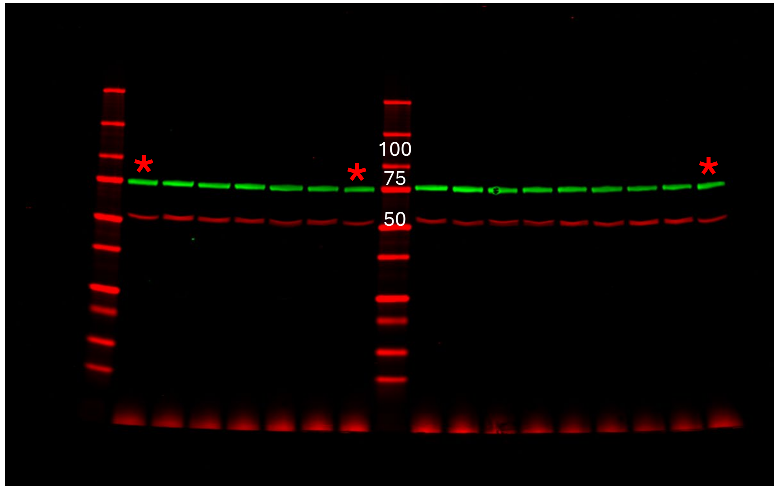

11 Gel Membrane Ab staining

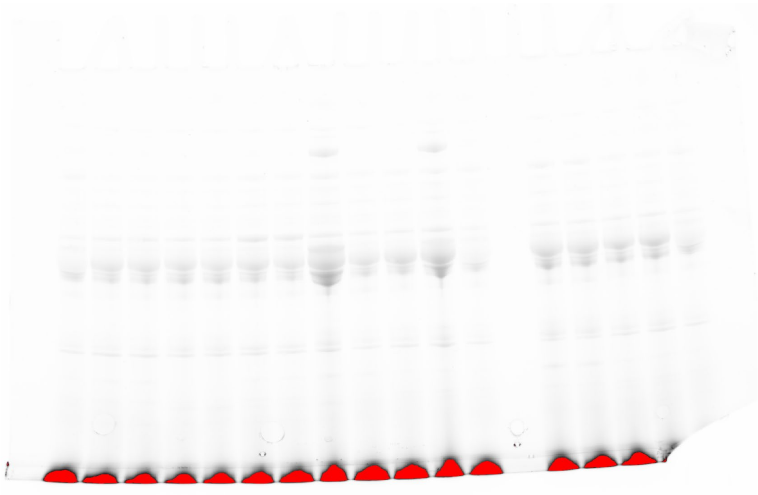

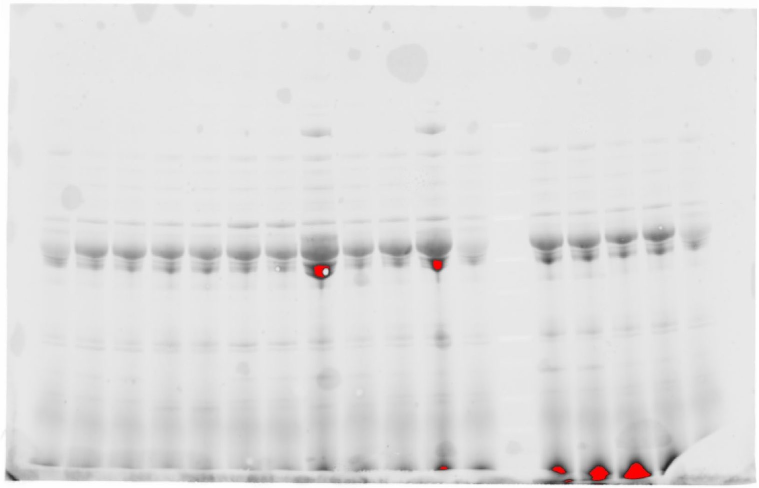

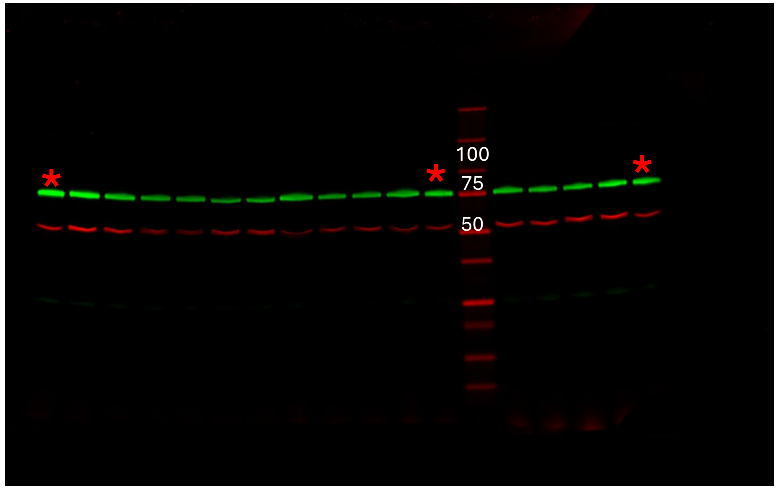

12

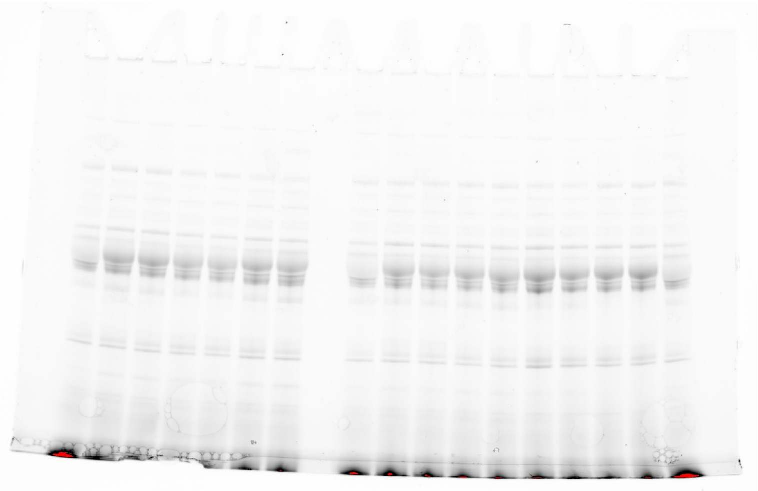

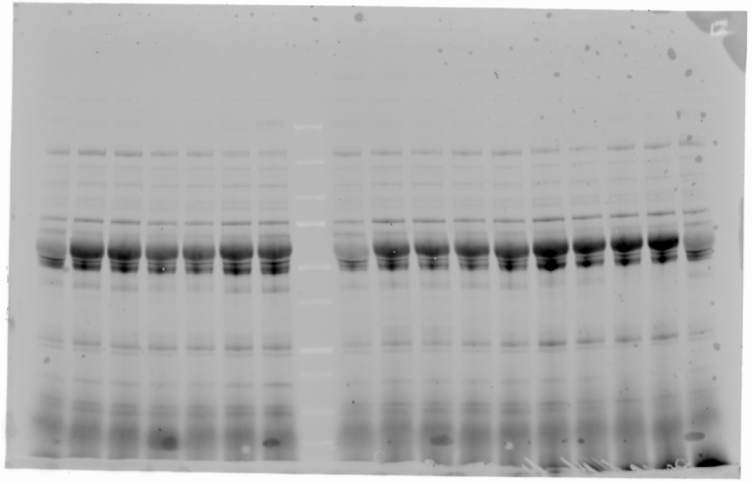

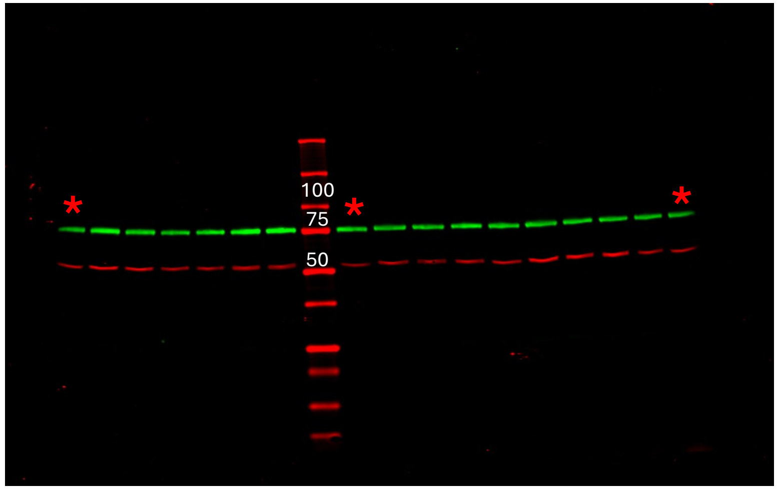

13 Gel Membrane Ab staining

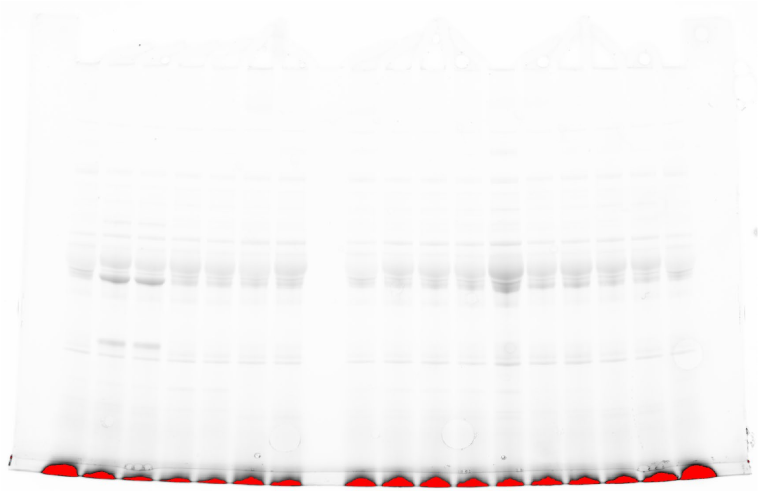

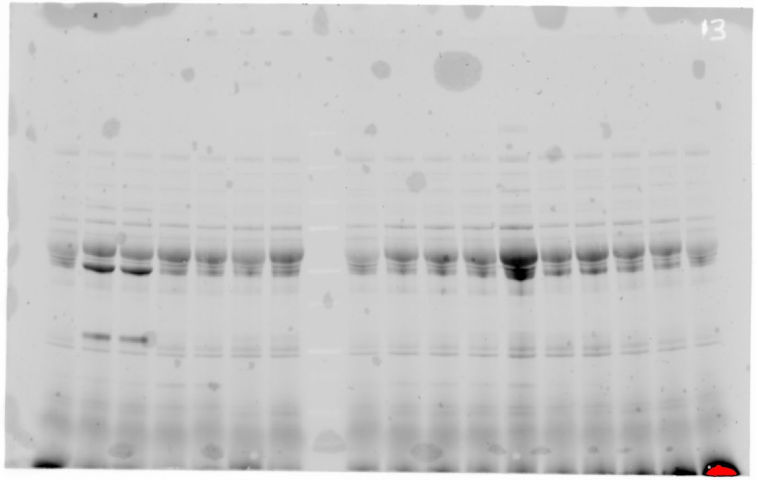

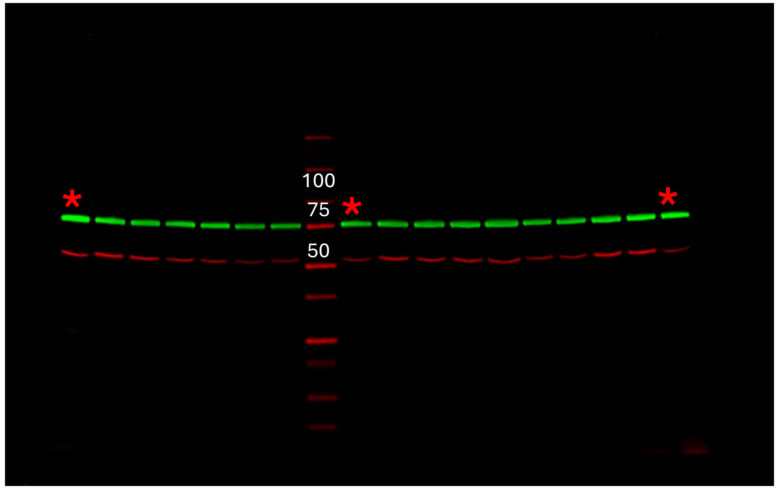

14

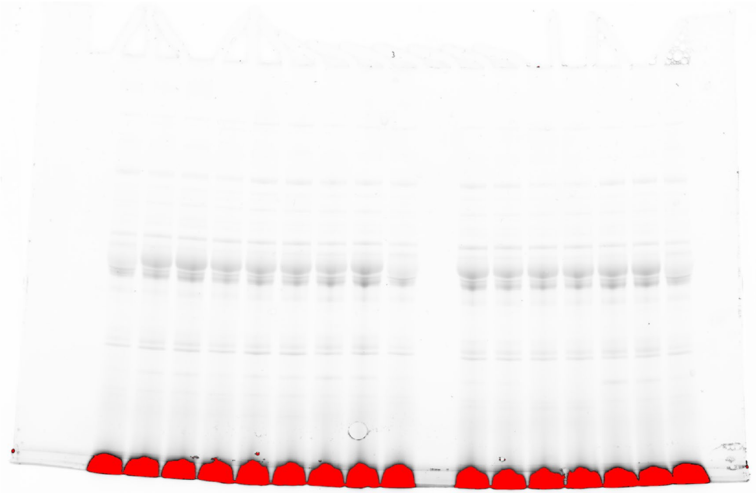

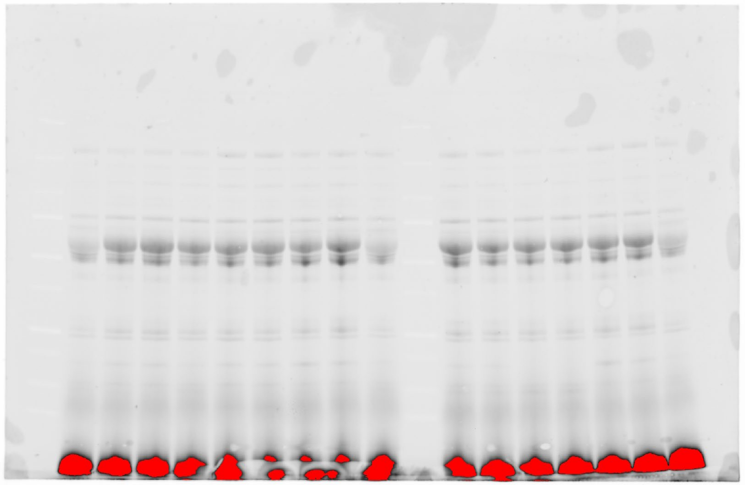

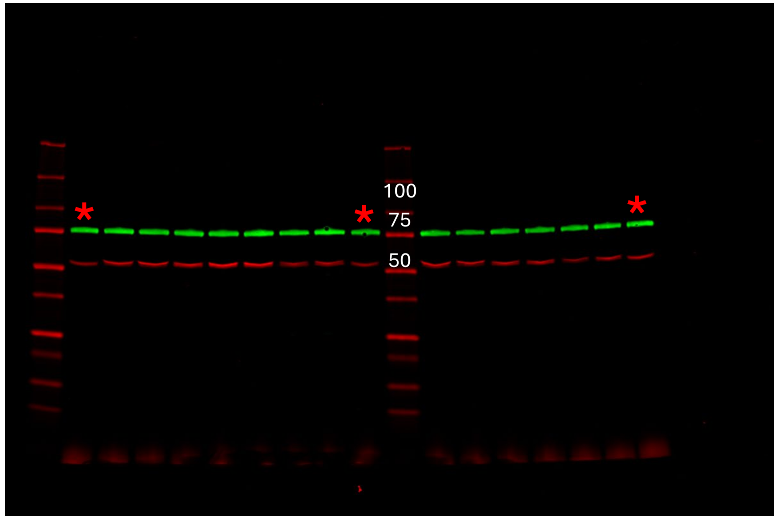

15 Gel Membrane Ab staining

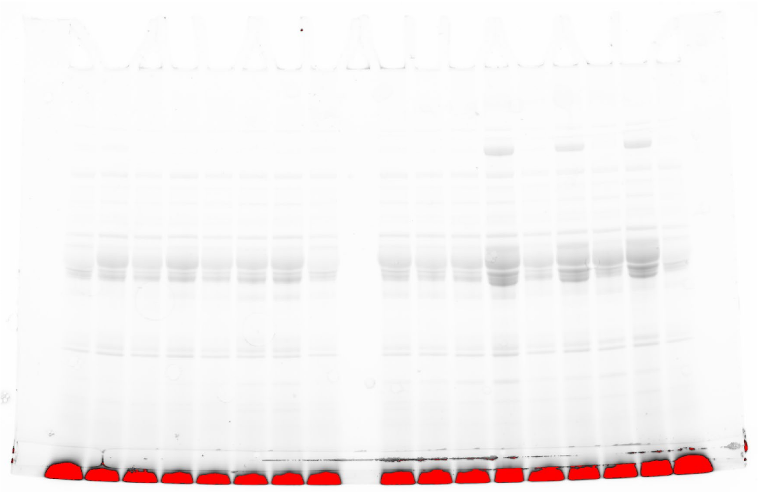

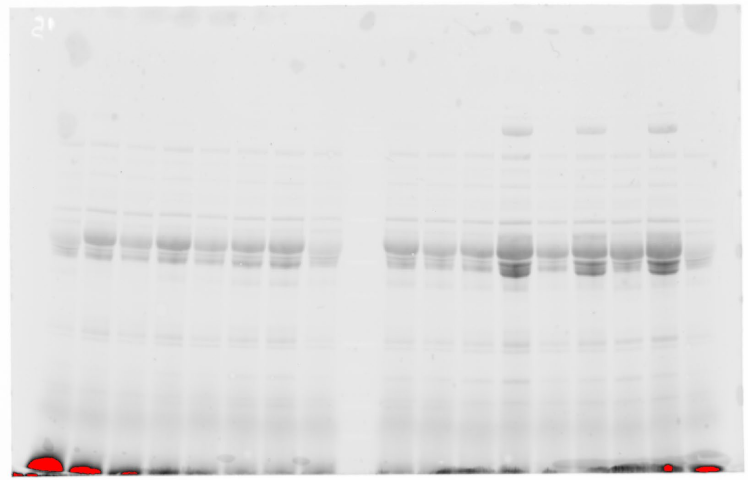

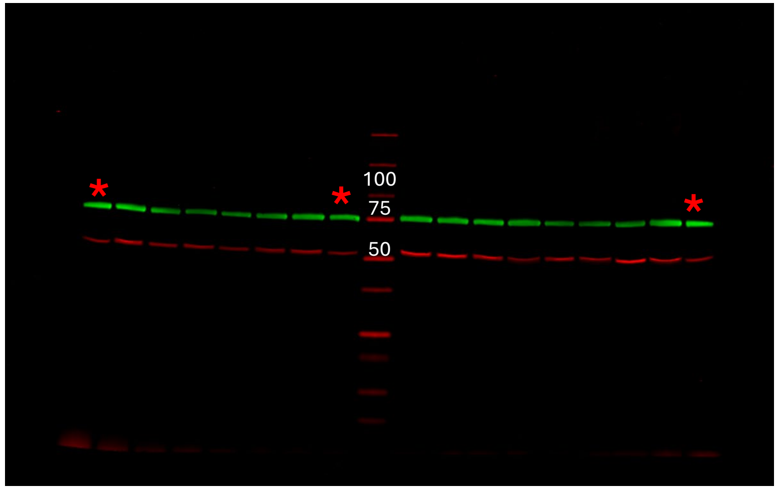

16

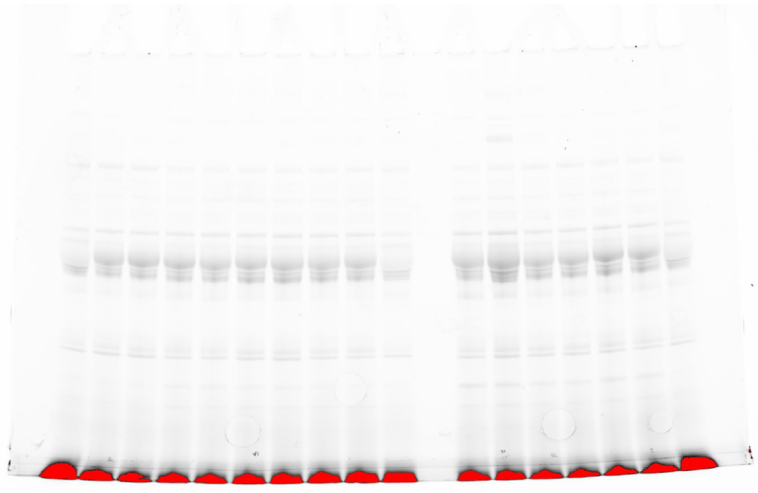

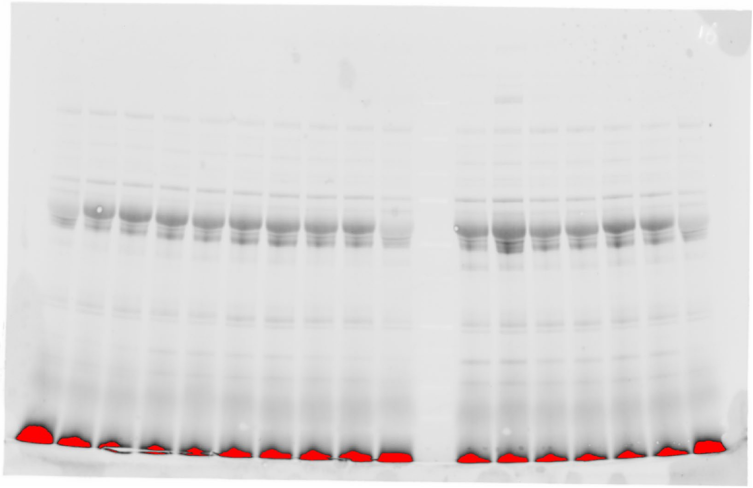

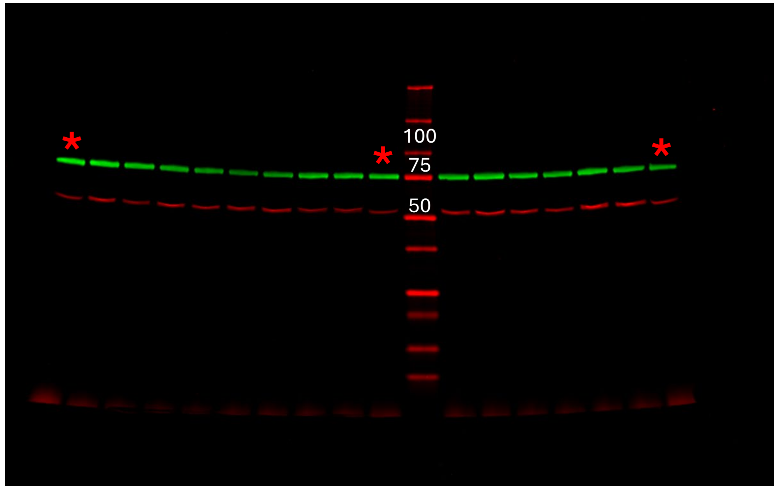

17

Gel

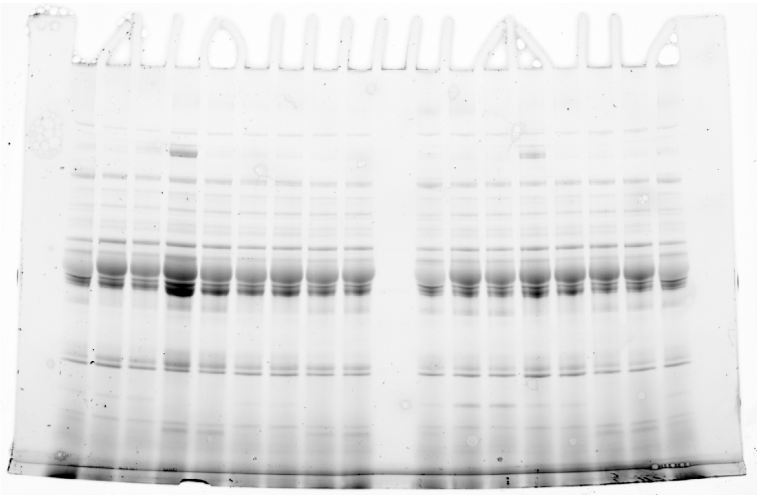

Membrane

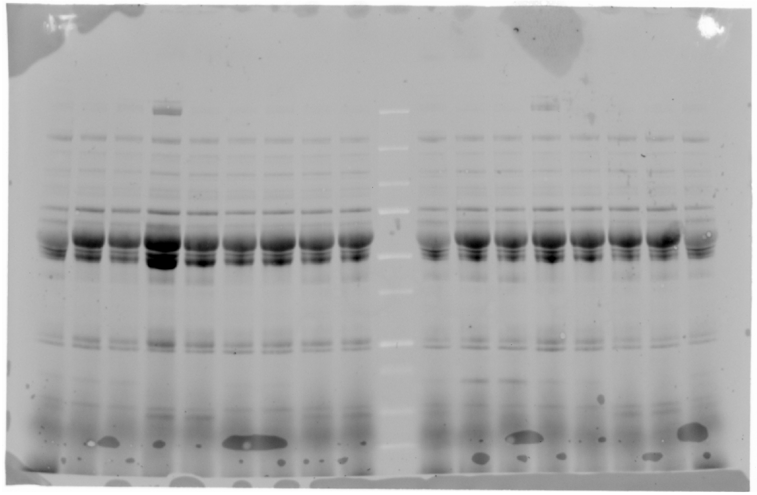

Ab staining

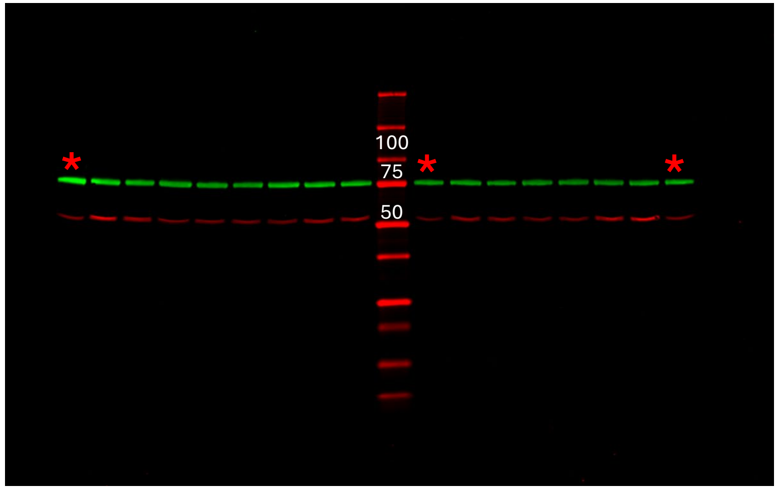

Supplement: Supplement 1 [file tvst-15-1-37_s001.pdf]
