# Supplementary material for: Circulating eNAMPT in Glaucoma: A Semi-Quantitative Plasma Analysis Before and After Nicotinamide Supplementation
Source: Transl Vis Sci Technol. 2026 Jan 28;15(1):37. doi: 10.1167/tvst.15.1.37 (PMC12859715; doi:10.1167/tvst.15.1.37)

Supplementary Figures:

Supplementary Figure 2. eNAMPT/Transferrin raw signals ratio of the reference sample depending on the position in the western blot assay. eNAMPT: extracellular nicotinamide phosphoribosyltransferase.

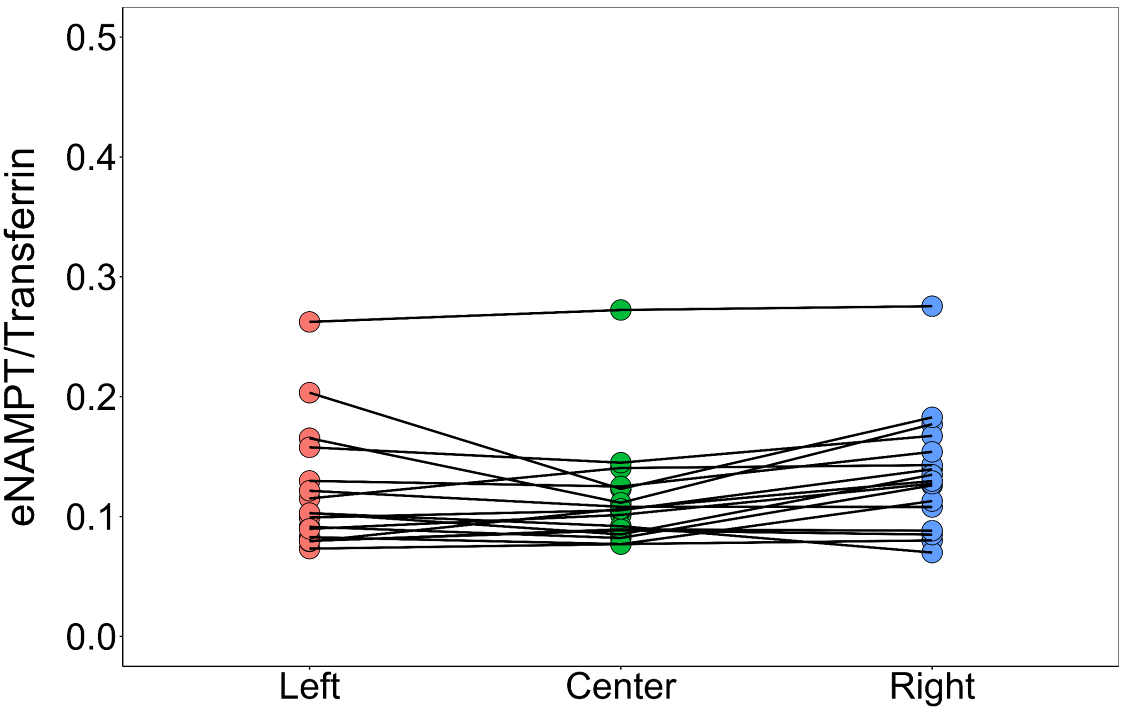

Supplementary figure 3. Heatmap representation of normalized relative extracellular nicotinamide phosphoribosyltransferase (eNAMPT) levels in plasma and their correlation with retinal vasculature parameters measured by optical coherence tomography angiography (OCTA) at baseline (pre-NAM treatment). Pearson correlation analyses were performed between plasma eNAMPT levels and regional OCTA perfusion metrics (FDR-adjusted p-value < 0.05 (*)). Pearson correlation coefficients are displayed within the tiles. No significant correlations were observed between eNAMPT levels and any of the measured vascular parameters. HTG: high-tension glaucoma; NTG: normal-tension glaucoma; ONH: optic nerve head; PEXG: pseudoexfoliative glaucoma.

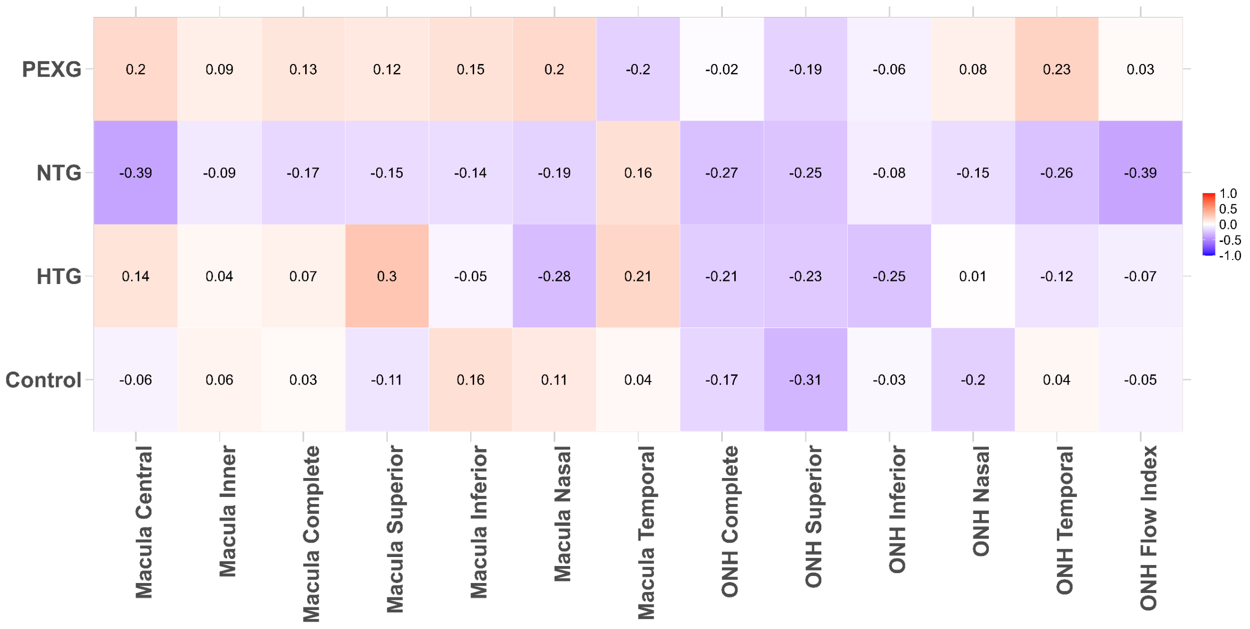

Supplementary figure 4. Heatmap representation of normalized relative extracellular nicotinamide phosphoribosyltransferase (eNAMPT) levels in plasma and their correlation with retinal vasculature parameters measured by optical coherence tomography angiography (OCTA) post-NAM treatment. Pearson correlation analyses were performed between plasma eNAMPT levels and regional OCTA perfusion metrics (FDR-adjusted p-value < 0.05 (*)). Pearson correlation coefficients are displayed within the tiles. No significant correlations were observed between eNAMPT levels and any of the measured vascular parameters. HTG: high-tension glaucoma; NTG: normal-tension glaucoma; ONH: optic nerve head; PEXG: pseudoexfoliative glaucoma.

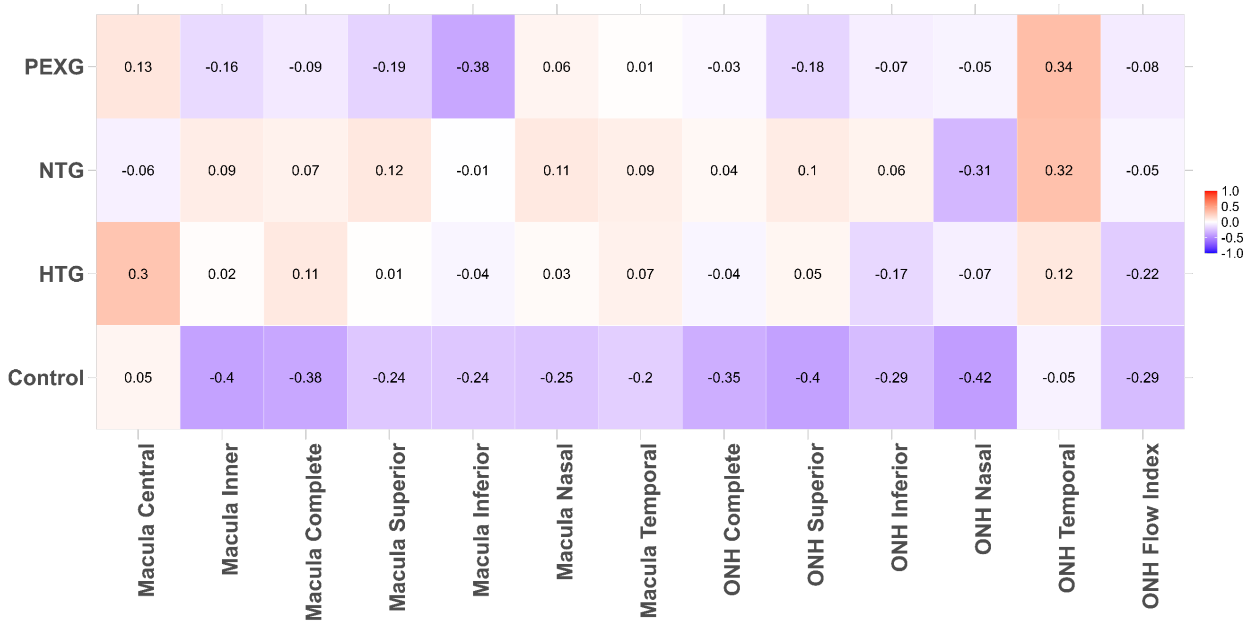

Supplement: Supplement 2 [file tvst-15-1-37_s002.pdf]
