# Supplementary material for: Circulating eNAMPT in Glaucoma: A Semi-Quantitative Plasma Analysis Before and After Nicotinamide Supplementation
Source: Transl Vis Sci Technol. 2026 Jan 28;15(1):37. doi: 10.1167/tvst.15.1.37 (PMC12859715; doi:10.1167/tvst.15.1.37)
Supplement: Supplement 3 [file tvst-15-1-37_s003.pdf]

Supplementary Table 1. Intra and inter-assay variability table. In the average raw signal columns and the average ratio column, the average from the respective reference sample triplicate in each blot for the band signals of extracellular nicotinamide phosphoribosyltransferase (eNAMPT), transferrin, and the ratio of their signals can be found. In the coefficient of variability (CV%) columns the CV% of extracellular nicotinamide phosphoribosyltransferase (eNAMPT) and transferrin, as well as the ratio of their signals for the reference sample is calculated within each blot. In bold the intra-assay CV% average between all blots can be found (14.9%) as well as the inter-assay CV% (37.9%).

		eNAMPT		Transferrin		eNAMPT/Transferrin	
		**Average raw signal**	**CV%**	**Average raw signal**	**CV%**	**Average ratio**	**CV%**
**Blot 1**		13062	9.1	163115	6.3	0.08	3.6
**Blot 2**		9171	4.2	87802	19.4	0.11	23.5
**Blot 3**		18866	16.3	167694	15.9	0.11	6.9
**Blot 4**		11862	16.5	71788	20.0	0.17	24.6
**Blot 5**		11832	14.4	107240	29.9	0.11	18.9
**Blot 6**		12395	7.4	93895	9.8	0.13	11.6
**Blot 7**		10119	2.7	120031	3.7	0.08	5.2
**Blot 8**		8621	15.2	90157	32.5	0.10	23.1
**Blot 9**		16310	5.6	157852	22.6	0.11	18.2
**Blot 10**		14458	13.0	92308	11.4	0.16	7.2
**Blot 11**		34230	22.3	252216	23.4	0.14	11.4
**Blot 12**		8732	1.0	60050	25.4	0.15	23.1
**Blot 13**		25056	39.9	279604	25.1	0.09	19.0
**Blot 14**		34917	6.7	129251	5.3	0.27	2.5
**Blot 15**		15015	5.5	175468	6.5	0.09	6.2
**Blot 16**		13850	4.8	163320	20.6	0.09	25.0
**Blot 17**		12128	13.8	121146	33.9	0.11	24.0
	**Average**	15919	11.7	137231	18.3	0.12	**14.9**
	**SD**	± 8093	± 9.4	± 60193	± 9.7	± 0.05	± 8.4
	**Min**	8621	1.0	60050	3.7	0.08	2.5
	**Max**	34917	39.9	279604	33.9	0.27	25.0
	**CV% (inter-assay)**	50.8		43.9		**37.9**	

Supplementary Table 2. Generalized linear mixed effects model (Gamma distribution with a log link and Gaussian random effects) including age and sex as co-variates. Age is rescaled by dividing it by 100. eNAMPT: extracellular nicotinamide phosphoribosyltransferase; HTG: high tension glaucoma; N Subject: number of levels of groups in the random effects, meaning number of unique participants included in the model; NAM: nicotinamide; NTG: normal tension glaucoma; PEXG: pseudoexfoliative glaucoma; Std: standard; τ Subject: variance of the random set intercepts associated with subject, representing variability within paired participants; σ^2: residual variance; (:) indicates interaction between two variables.

	**Normalized relative eNAMPT levels**		
Predictors	*Estimate*	*Std. error*	*P-value*
Controls (Intercept)	0.12730	0.51163	0.804
HTG	-0.17101	0.11632	0.142
NTG	-0.11845	0.11648	0.309
PEXG	-0.01916	0.11635	0.869
NAM Treatment	-0.02334	0.03212	0.467
Age	0.23485	0.68110	0.730
Sex (Male)	0.08061	0.08064	0.317
HTG : NAM Treatment	0.01982	0.04586	0.666
NTG : NAM Treatment	-0.01136	0.04592	0.805
PEXG : NAM Treatment	-0.08570	0.04618	0.063
**Random Effects**			
σ^2	0.02841		
$\tau_{Subject}$	0.06		
$N_{Subject}$	120		
Observations	236		

Supplementary Table 3. Descriptive statistics of the extracellular nicotinamide phosphoribosyltransferase (eNAMPT) relative normalized values in the different study groups (overall, sex dependent) at baseline (Pre-NAM) and after nicotinamide (NAM) treatment (Post-NAM) corresponding to the boxplot values illustrated in Figures 2 and 3B. HTG: high tension glaucoma; NTG: normal tension glaucoma; PEXG: pseudoexfoliative glaucoma.

Group		N	Median	Q1	Q3	Lower Whisker	Upper Whisker
	**Baseline (Pre-NAM)**						
**Control**	Overall	30	1.40	1.17	1.81	0.64	2.37
	Male	16	1.31	0.97	1.65	0.64	2.37
	Female	14	1.61	1.33	1.98	1.17	2.17
**HTG**	Overall	30	1.34	1.04	1.55	0.52	1.82
	Male	15	1.33	1.04	1.56	0.85	1.81
	Female	15	1.35	1.19	1.54	0.98	1.82
**NTG**	Overall	30	1.35	1.03	1.55	0.54	2.19
	Male	15	1.34	1.04	1.51	0.96	1.64
	Female	15	1.35	1.03	1.57	0.54	2.19
**PEXG**	Overall	29	1.42	1.26	1.66	0.79	1.98
	Male	15	1.54	1.39	1.78	1.06	1.95
	Female	14	1.27	1.06	1.42	0.79	1.74
	**NAM Treatment (Post-NAM)**						
**Control**	Overall	30	1.47	1.07	1.69	0.55	2.51
	Male	16	1.36	1.04	1.82	0.55	2.51
	Female	14	1.53	1.34	1.67	1.07	1.93
**HTG**	Overall	29	1.39	1.12	1.62	0.49	2.24
	Male	15	1.39	0.89	1.71	0.78	2.10
	Female	14	1.37	1.21	1.53	1.16	1.65
**NTG**	Overall	29	1.28	0.99	1.46	0.63	1.85
	Male	14	1.28	1.03	1.46	0.67	1.58
	Female	15	1.28	0.94	1.47	0.63	1.85
**PEXG**	Overall	29	1.24	1.02	1.48	0.70	1.97
	Male	16	1.45	1.29	1.79	1.15	1.97
	Female	13	0.97	0.87	1.15	0.70	1.42
